# Septins Are Important for Cell Polarity, Septation and Asexual Spore Formation in *Neurospora crassa* and Show Different Patterns of Localisation at Germ Tube Tips

**DOI:** 10.1371/journal.pone.0063843

**Published:** 2013-05-14

**Authors:** Adokiye Berepiki, Nick D. Read

**Affiliations:** 1 Biosciences, University of Exeter, Exeter, United Kingdom; 2 Fungal Cell Biology Group, Institute of Cell Biology, University of Edinburgh, Edinburgh, United Kingdom; Dartmouth College, United States of America

## Abstract

Septins are GTP-binding cytoskeletal proteins that contribute to cell polarity, vesicle trafficking, cytokinesis and cell morphogenesis. Here we have characterised the six septins encoded by the genome of the model filamentous fungus *Neurospora crassa*. Analysis of septin null mutants demonstrated that septins limit the sites of emergence of germ tubes and are important for septation and conidiation in *N. crassa*. Septins constituted a range of different higher-order structures in *N. crassa* – rings, loops, fibres, bands, and caps – which can co-exist within the same cell. They showed different patterns of localisation at germ tube tips, with GFP-CDC-10 and CDC-11-GFP forming a subapical collar with lower signal intensity at the tip apex, CDC-3-GFP and CDC-12-GFP organized as a cap at the tip apex and GFP-ASP-1 forming an extended subapical collar. Purification of the septin complex and mass spectrometry of isolated proteins revealed that the septin complex consists predominantly of CDC-3, CDC-10, CDC-11 and CDC-12. Immunoprecipitation of the putative septin ASP-1 revealed that this protein interacts with the core septin complex.

## Introduction

Septins are found in a range of eukaryotes from animals to yeasts but are absent from higher plants and certain protists [1], [2]. The septins were first discovered in the budding yeast *Saccharomyces cerevisiae* in a mutant screen by Hartwell and colleagues [3]. They are a family of GTP-binding proteins that interact with actin, microtubules and membranes and are involved in regulating cytoskeletal dynamics, secretion and membrane remodelling [4]. Septins form polarised, highly organised, oligomeric filaments which assemble into a variety of higher order (HO) structures [5]–[7]. The incorporation of a single alternative subunit, which in turn can be regulated by phosphorylation, allows the formation of different HO assemblies [8].

*In vitro*, septins purified from Drosophila, mammals, and budding yeast form short (∼32 nm long) filaments, which can assemble end to end into >1500 nm long paired filaments upon dialysis into physiological (75 mM) salt [9]. Purified mammalian septins self-assemble into rings 0.6 µM in diameter and in the presence of the adaptor protein anillin organises actin filaments into bundles while recruiting septin filaments to the bundles [10]. Ultrastructural analysis of yeast sphaeroplasts has demonstrated that septins are found as rings and as ordered gauzes at the cell cortex [11].

In the budding yeast, septins demarcate areas of the plasma membrane and scaffold proteins, which are crucial for the proper assembly of protein complexes such as the contractile actomyosin ring (CAR) and polarisome [12]–[15]. The bud ring acts as a diffusion barrier for polarisome components in the bud. During cytokinesis the septin ring scaffolds the CAR and related cytokinetic proteins and acts as a diffusion barrier to maintain diffusible exocyst and polarisome proteins at the cleavage site [12]–[14]. Septins have been shown to limit diffusion of plasma membrane protein-encoding mRNA (*Ist2*) thereby compartmentalising the plasma membrane [16]. Additionally, a septin-dependent diffusion barrier forms in the nuclear envelope and limits the movement of extant nuclear pores into the bud [17]. The barrier also contributes to the segregation of other ageing factors, such as carbonylated proteins and DNA circles, thereby ensuring the asymmetric inheritance of age during cell division [17].

Septins have been well studied in budding yeast and to a lesser extent in the fission yeast, *Schizosaccharomyces pombe*. Filamentous fungi, however, provide a range of cell types and morphologies and tractable experimental systems with which to decipher the roles of septins and elucidate novel functions [18]. Unlike budding yeast, various HO septin assemblies can coexist within hyphae of the filamentous fungus *Ashbya gossypii* and these structures utilise different signalling pathways for their assembly and maintenance [7]. For example deletion of Gin4 or Elm1 kinases completely abolished the formation of inter-region septin rings (IR rings - septin rings in the middle of a hyphal cell) but not tip-associated septin filaments or septin rings at branch points [7].

In the trimorphic pathogen *C. albicans*, *cdc3* and *cdc12* are essential whereas Δ*cdc10*/Δ*cdc10* and Δ*cdc11*/Δ*cdc11* null mutants were viable but displayed conditional defects in cytokinesis, cell wall deposition and bud morphology [19]. *Candida albicans* Δ*cdc10*/Δ*cdc10* and Δ*cdc11*/Δ*cdc11*deletion mutants formed abnormal hyphae and the Δ*cdc11*/Δ*cdc11* and Δ*cdc10*/Δ*cdc10* null mutants were defective for invasive growth and virulence [19], [20]. Interestingly in *C. albicans*, septin localisation in hyphae is different from that observed during budding or pseudohyphal growth. Septins localise to a tight ring at the bud and pseudohyphal necks whereas in emerging germ tubes septins localise as a diffuse collar or cap at the site of outgrowth [19]. Additionally, a faint cap of septins is also present at the tips of *C. albicans* hyphae but not pseudohyphae [19]. Analysis of septin gene expression in *C. albicans* has demonstrated that none of the septins are hyphal specific, which suggests that the change in septin localisation is due to changes in septin regulation during morphogenetic transitions [19].

In the dimorphic plant pathogen *Ustilago maydis* none of the four septin genes are essential, however, all single septin deletion mutants show conditional lethality when grown at 34°C [21]. At restrictive temperatures septin null mutants were swollen and regularly lysed; both defects could be rescued by the addition of sorbitol [21]. Additionally, septin deletion mutants were hypersensitive to compounds known to affect cell wall integrity (caffeine, calcofluor white and chlorpromazine) suggesting that the primary defect in these mutants is in cell wall construction and possibly osmoregulation [21]. Septins in *U. maydis* were also shown to have roles in infection [21], [22] similar to septins in *C. albicans* and *Cryptococcus neoformans* [20], [23]. The septins formed three HO structures in *U. maydis*: bud neck collars, band-like structures at the growing hyphal tip and long septin fibres that partially colocalised with microtubules [21]. Only Sep4 (*S. cerevisiae cdc10* ortholog) formed long septin fibres in *U. maydis* [21]. Cdc10 septin fibres have also been shown to occasionally colocalise with microtubules (MTs) in dikaryotic hyphae of *C. neoformans* [23]. Interestingly in the plant pathogen *Magnaporthe oryzae*, the location of the appressorium septum is determined by the site of septin ring assembly [24]. During appressorium formation the septin ring functions as both a scaffold for Tea-1-mediated F-actin assembly and as a diffusion barrier for the Rvs167 I-BAR protein, which is thought to induce membrane curvature at the tip of the emerging penetration peg [25].

The genome of filamentous fungus *Aspergillus nidulans* encodes a complement of five septins, four of which, *aspA*, *aspB*, *aspC*, *aspD*, are orthologous to the *S. cerevisiae* septins *cdc11*, *cdc3*, *cdc12* and *cdc10*, respectively. The *aspE* gene is a member of group 5 septins and orthologs are restricted to other filamentous fungi. All five septins are expressed during vegetative growth, demonstrating a lack of sporulation-specific septins, with *aspB* (*cdc3* ortholog) having the highest expression level [26]. Deletion of *aspA*, *aspB*, or *aspC* in *A. nidulans* leads to delayed septation, increased emergence of germ tubes and hyphal branches and decreased asexual sporulation [27], [28]. These findings suggest that one of the key function of septins in filamentous fungi is to prevent the inappropriate emergence of germ tubes and branches, which is in keeping with their known role as a diffusion barrier and scaffold in budding yeast; presumably cell polarity components are concentrated at a specific site by HO septin structures and are prevented from diffusing freely. Defects in conidiation and septation in septin null mutants demonstrate that septins play a minor role in cytokinesis. In *A. nidulans* and *A. fumigatus,* septins localise as rings in forming septa and emerging branches and germ tubes [27]–[29].

To further understand the role of septins in filamentous fungi we analysed the entire septin complement of the model filamentous fungus *Neurospora crassa* using deletion mutants, live-cell imaging and mass spectrometry of purified septin complexes. The genome of *N. crassa* encodes six putative septins [2]. Four ORFS (NCU8207, NCU3515, NCU2464 and NCU3795) are orthologs of the core *S. cerevisiae* septins, *cdc3*, *cdc10*, *cdc11*, and *cdc12* and two hypothetical proteins (ORFs NCU1998 termed *asp-1* and NCU6414 termed *asp-2*) contain canonical septin sequences but are only found in filamentous fungi (Pan et al., 2007).

Here we show that the *N. crassa* septins CDC-3, CDC-10, CDC-11, and CDC-12 participate in septation, cell polarity, and conidiation. Septins formed a range of HO assemblies: rings, loops, fibres, bands and caps. Intriguingly, septins displayed distinct patterns of localisation in germ tube tips suggesting they may perform different roles during tip growth. Mass spectrometry analysis of purified septin complexes revealed that the core complex consisted predominately of CDC-3, CDC-10, CDC-11, and CDC-12, with ASP-1 only detectable in immunoprecipitated complexes when ASP-1 was used as a bait. We were unable to detect a phenotype for the *asp-2* null mutant, localise GFP-ASP-2 or find evidence of its expression during vegetative growth.

## Results

### Septins Limit the Emergence of Germ Tubes and are Involved in Conidiation

**Figure 1:**
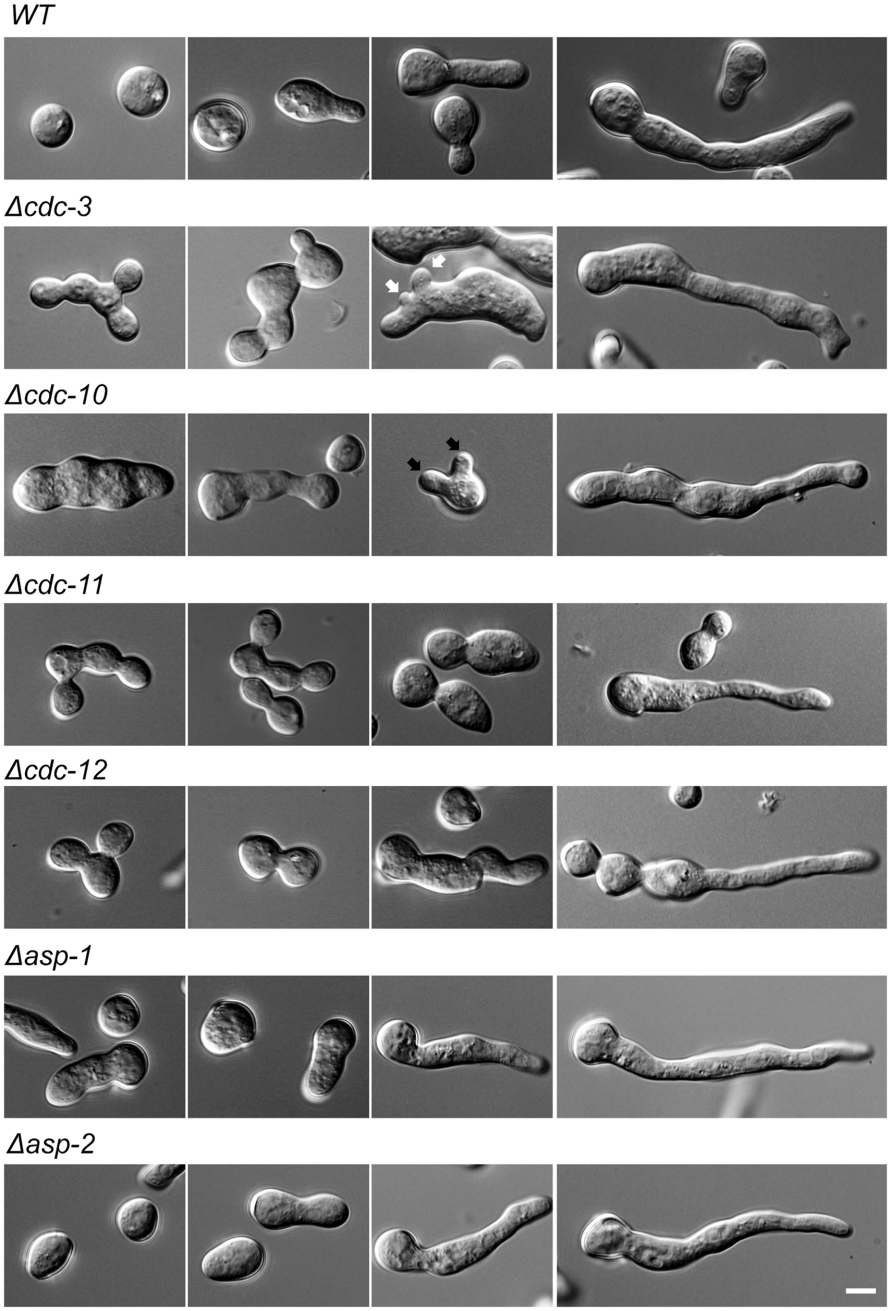
Septin deletion strains have altered morphologies. Wild-type and single septin deletion strains were incubated in liquid VMM for up to 5 h then imaged by DIC microscopy to obtain representative images of germination and development. Septin deletion strains, with the exception of *Δasp-1* and *Δasp-2*, fail to properly separate conidia and often appeared swollen and misshapen. Black arrows denote multiple germ tube emergence. White arrows show multiple CAT emergence. Scale bar, 5 µm.

**Figure 2:**
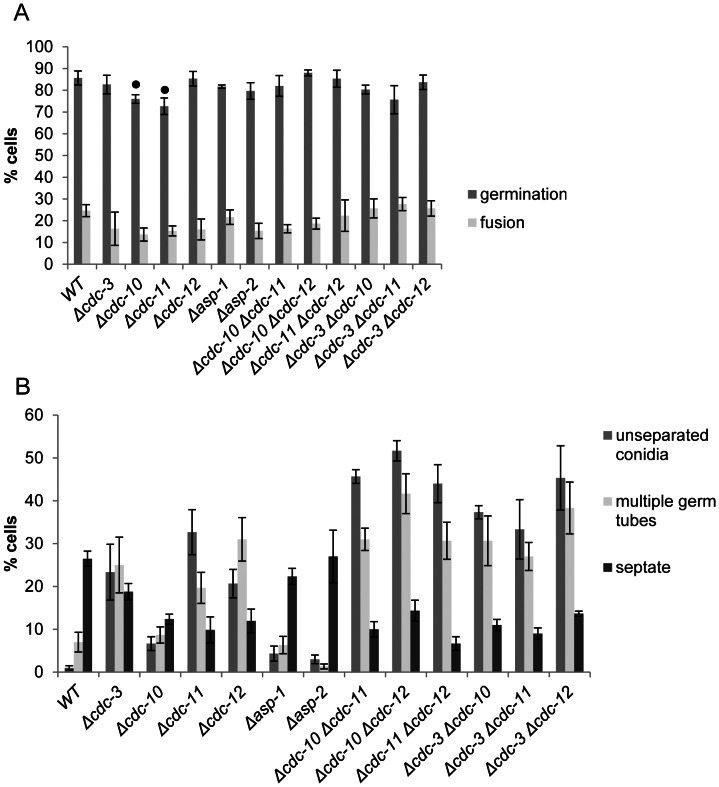
Septin deletion strains display an increase in germ tube emergence and unseparated conidia and a reduction in septation compared to wild-type. (**A**) The number of unseparated cells in was counted before incubation, the number of cells with more than two germ tubes was counted following 3 h of incubation in liquid VMM and the number of septate cells was determined with DIC microscopy after 5 h of incubation in liquid VMM (*n* = 300). There was a significant increase (p = <0.05) in the percentage of unseparated conidia and cells with multiple germ tubes compared to wild-type for all strains with the exception of Δ*cdc10*, Δ*asp-1* and Δ*asp-2*. Similarly, there was a significant decrease (p = <0.05) in the percentage of septate cells for all strains compared to the wild-type except for Δ*asp-1* and Δ*asp-2*. (**B**) Strains were incubated in liquid VMM for 3 h then quantified for germination and cell fusion (*n* = 300). A black dot above bars indicate a significance difference (p = <0.05) compared to the wild-type control. The germination rates of Δ*cdc-10* and Δ*cdc-11* were slightly reduced compared to the wild-type.

Bioinformatic and phylogenetic analysis of the *N. crassa* genome has revealed the presence of six putative septins [2]. Four ORFS (NCU8207, NCU3515, NCU2464 and NCU3795) are orthologs of the core *S. cerevisiae* septins, *cdc3*, *cdc10*, *cdc11*, and *cdc12*, respectively, so we adopted this nomenclature as recommended by Pan et al. (2007). Two hypothetical proteins were found to contain canonical septin sequences but are only found in filamentous fungi [2]. These two ORFs, NCU1998 and NCU6414, were named ASP-1 and ASP-2, respectively. To determine the role of septins in *N. crassa* we analysed strains in which individual septins were deleted or where a combination of core septins were deleted. Previous studies in *A. nidulans* have shown that septins delay and limit the emergence of germ tubes and are involved in septation [28]. To understand if this is a general function of septins in filamentous fungi we initially assessed the rates of germination and cell fusion, septation and the morphology of septin null mutants and compared these with the wild type (Figures 1 and 2). A slight but significant (p = <0.05) decrease in the rate of germination in Δ*cdc-10* and Δ*cdc-11* strains was observed, which suggests that in these strains germ tubes emerge later than in the wild-type. No significant differences in germination and fusion rates were observed for *cdc-3*, *cdc-12*, *asp-1* and *asp-2* deletion mutants (Figure 2A).

**Figure 3:**
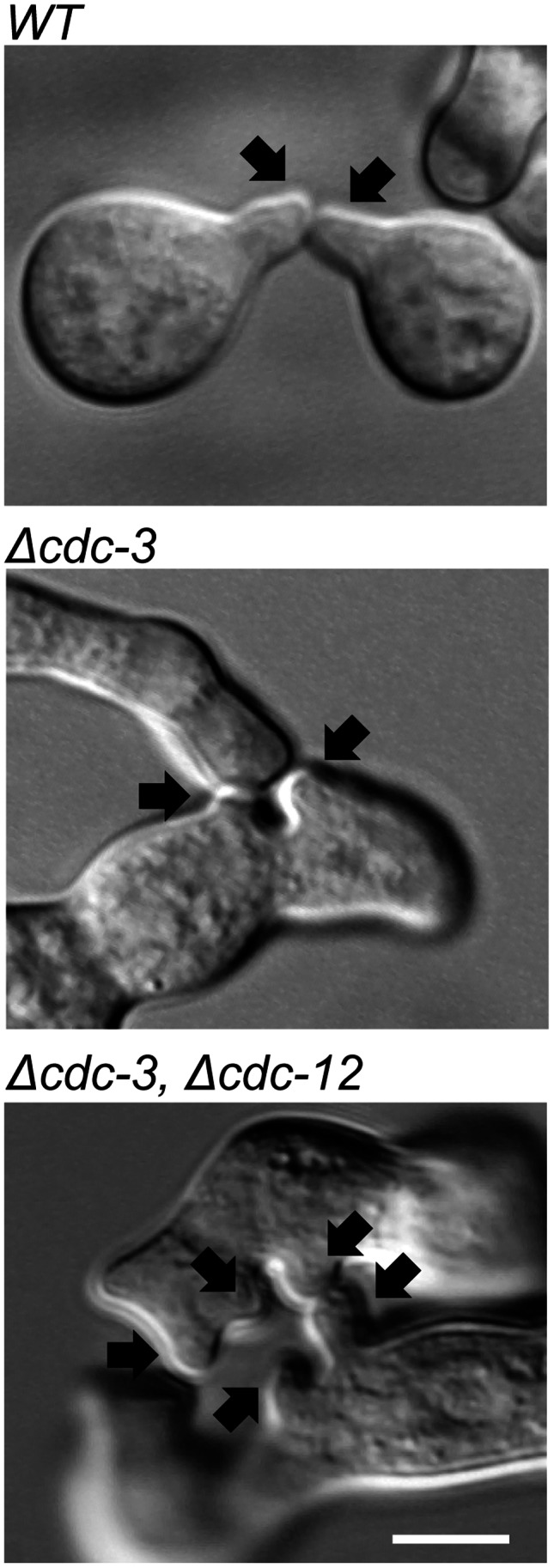
CAT-mediated cell fusion is affected in septin null mutants. Wild-type and single septin deletion strains were incubated in liquid VMM for up to 5 h then imaged by DIC microscopy to obtain representative images of cell fusion. In the wild-type each conidium most commonly formed a single CAT. In septin deletion strains, often several CATs are formed during cell fusion. Black arrows denote individual CATs prior to fusion. Scale bar, 5 µm.

Despite the relatively minor effects of the absence of individual septins on germination and fusion rates, the morphologies of the *cdc-3*, *cdc-10*, *cdc-11*, and *cdc-12* deletion strains were markedly different from that of the wild-type. There were no obvious morphological defects for Δ*asp-1* and Δ*asp-2* strains (Figure 1). The major morphological defects displayed by strains lacking core septins included joined conidia, swelling, and multiple germ tube and CAT emergence (Figures 1 and 3). The presence of joined conidia was probably due to defects during conidiation and most likely stems from a failure of conidia to separate during budding due to the lack of a functional septin ring. We quantified the amount of conidia that were unseparated or exhibited more than one site of germ tube outgrowth and found a significant (p = <0.05) increase in Δ*cdc-3*, Δ*cdc-10*, Δ*cdc-11*, and Δ*cdc-12* strains compared to wild-type (Figure 2). Strains lacking the core septins displayed a significant (p = <0.05) increase in the number of cells producing more than one germ tube and the proportion of unseparated cells than the wild-type, although the phenotype of the *cdc-10* null mutant was not as severe. The Δ*asp-1* and Δ*asp-2* strains, however, were similar to wild-type.

**Figure 4:**
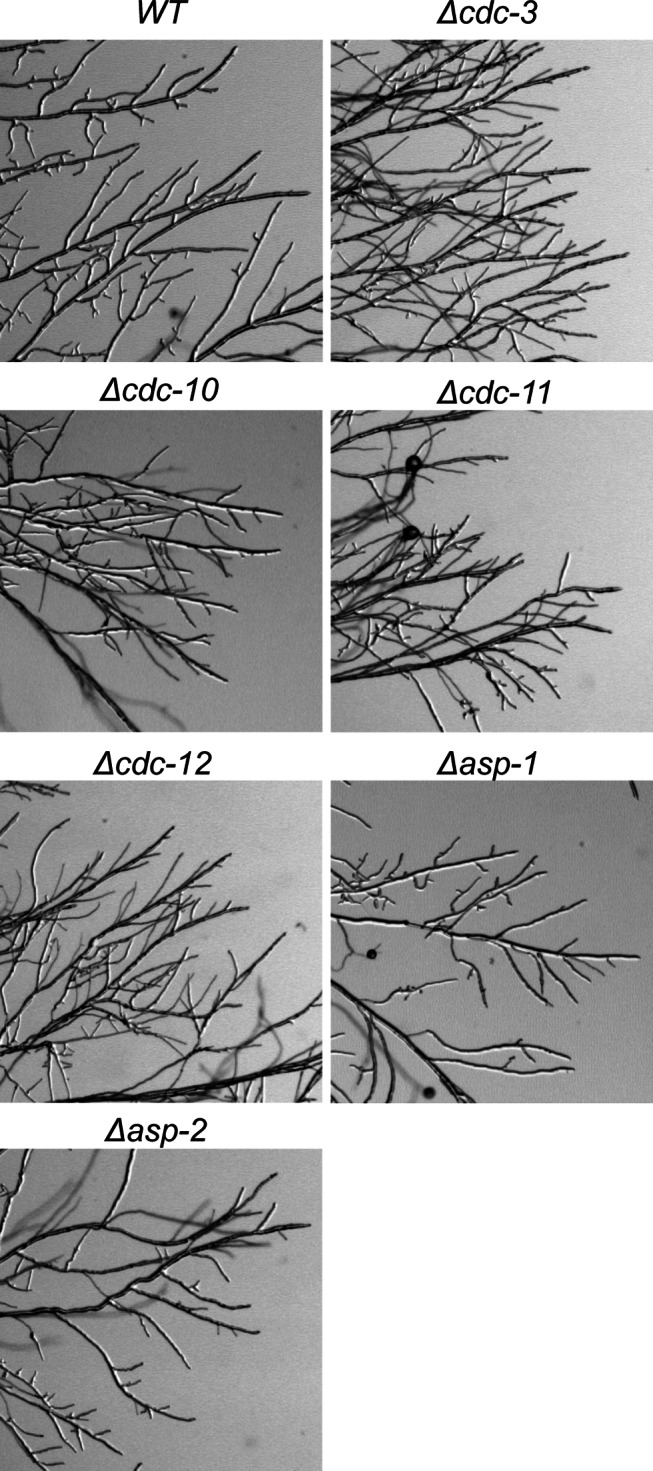
Branching is more prevalent at the colony edge in septin null mutants. Wild-type and septin deletion strains were inoculated onto solid VMM, incubated for 18 d at 35°C then imaged using a stereomicroscope. Septin deletion strains, with the exception of *Δasp1-1* and *Δasp-2*, appeared to form more branches at the colony edge.

**Figure 5:**
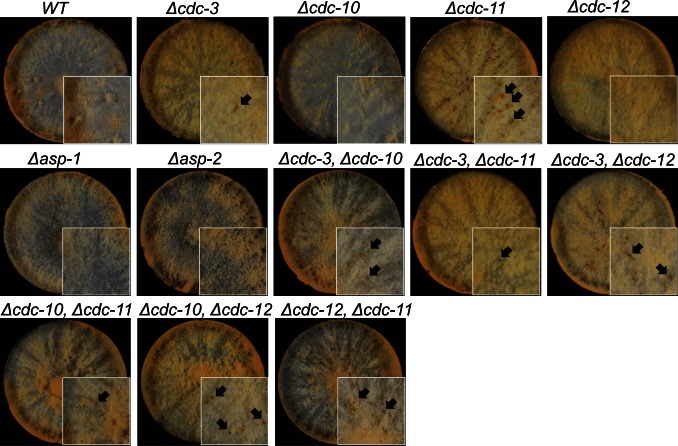
Colony morphology is altered in septin deletion strains. Wild-type and septin deletion strains were inoculated onto solid VMM, incubated for 3 d at 24°C then photographed. Septin deletion strains, with the exception of *Δasp1-1* and *Δasp-2*, formed denser colonies, did not produce as many aerial hyphae as wild-type and occasionally regions of cell lysis were visible (black arrows).

We reasoned that an increase in sites of germ tube outgrowth might be accompanied by a reduction in radial extension due to hyperbranching so we measured the radial extension rates of the septin mutants and imaged branching in the mature colony (Table 1 and Figure 4). Indeed, strains lacking the core septins appeared to form more branches at the colony edge and showed a marked reduction in radial extension compared to the wild-type with Δ*cdc-3* colonies extending at a rate that was 25% slower than the wild-type. Again the Δ*cdc-10* strain showed a less severe phenotype, with an extension rate only 5% slower than wild type. The colony morphologies of Δ*cdc-3*, Δ*cdc-11*, and Δ*cdc-12* were slightly denser than the wild-type and showed areas of cell lysis whereas the Δ*cdc-10*, Δ*asp-1*, and Δ*asp-2* mutants appeared unaffected (Figure 5). Given the role for septins in septation and cytokinesis we assessed whether septation still occurred in the deletion mutants and if so whether there was any reduction in their formation. We found that septa still formed in the mutants and appeared identical to wild-type septa suggesting that septins are not required for septum formation *per se*; however, there was a significant reduction (p = <0.05) in the number of septate germ tubes formed after 5 h in the Δ*cdc-3*, Δ*cdc-10,* Δ*cdc-11* and Δ*cdc-12* mutants suggesting that septation is delayed. Research in *U. maydis* and *C. albicans* has shown that in the absence of septins the cell wall is abnormal. However, calcofluor white staining of *N. crassa* septin deletion mutants did not reveal any defects in cell wall formation at septa or the cell envelope (Figure S1).

**Table 1:** Radial extension of *N. crassa* septin mutants.

Strain	Extension rate±S.d.(mm/hour)*
*WT*	2.4±0.6
*Δcdc-3*	1.8±0.4
*Δcdc-10*	2.2±0.4
*Δcdc-11*	1.7±0.6
*Δcdc-12*	1.7±0.4
*Δasp-1*	2.5±0.5
*Δasp-2*	2.4±0.4
*Δcdc-10 Δcdc-11*	1.6±0.3
*Δcdc-10 Δcdc-12*	1.7±0.4
*Δcdc-11 Δcdc-12*	1.7±0.4
*Δcdc-3 Δcdc-10*	1.8±0.3
*Δcdc-3 Δcdc-11*	1.6±0.4
*Δcdc-3 Δcdc-12*	1.6±0.4

* The radial extension of mycelia was assessed after 8 h growth on VMM plates at 35°C. Colony extension was measured along four randomly chosen radii on duplicate plates; *n* = 8 for each strain.

**Figure 6:**
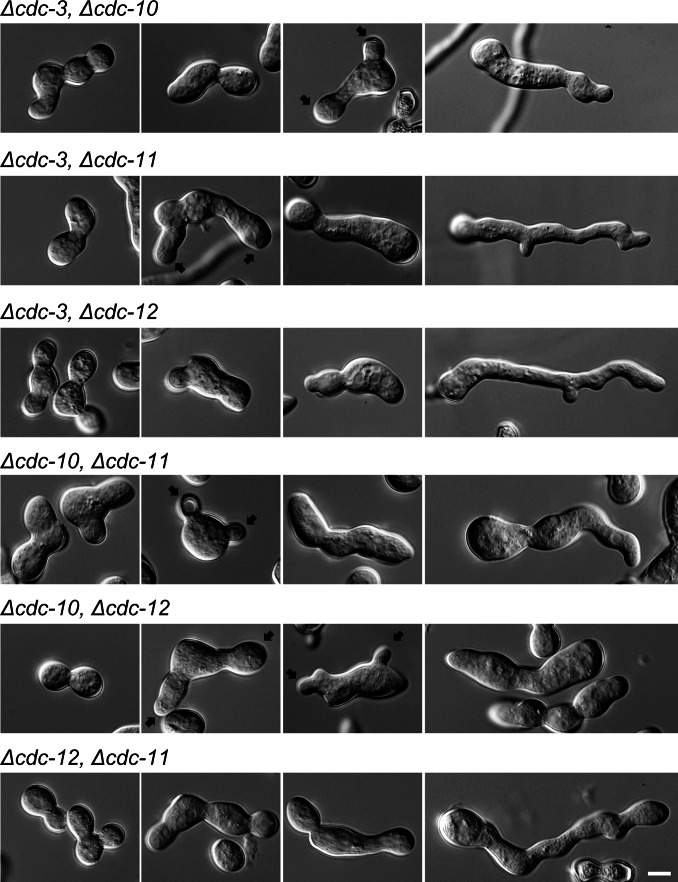
Septin double deletion strains have altered morphologies. Wild-type and septin deletion strains were incubated in liquid VMM for up to 5 h then imaged by DIC microscopy to obtain representative images of germination and development. Septin double deletion strains were swollen and misshapen and failed to properly separate conidia. Black arrows denote multiple germ tube emergence. Scale bar, 5 µm.

To further dissect the functional roles of the *N. crassa* core septins we constructed double mutants lacking two core septins in different combinations. The resulting strains were all viable and exhibited similar germination and fusion rates to the wild-type (Figure 2A). Cell and colony morphologies of the double deletion strains were comparable to the single null mutants (Figures 5 and 6). However, the amount of unseparated cells and germ tubes was significantly (p = <0.05) increased and septation significantly reduced (p = <0.05) in the double mutants compared to single deletion strains suggesting that individual core septins may have non-overlapping roles (Figure 2B).

**Figure 7:**
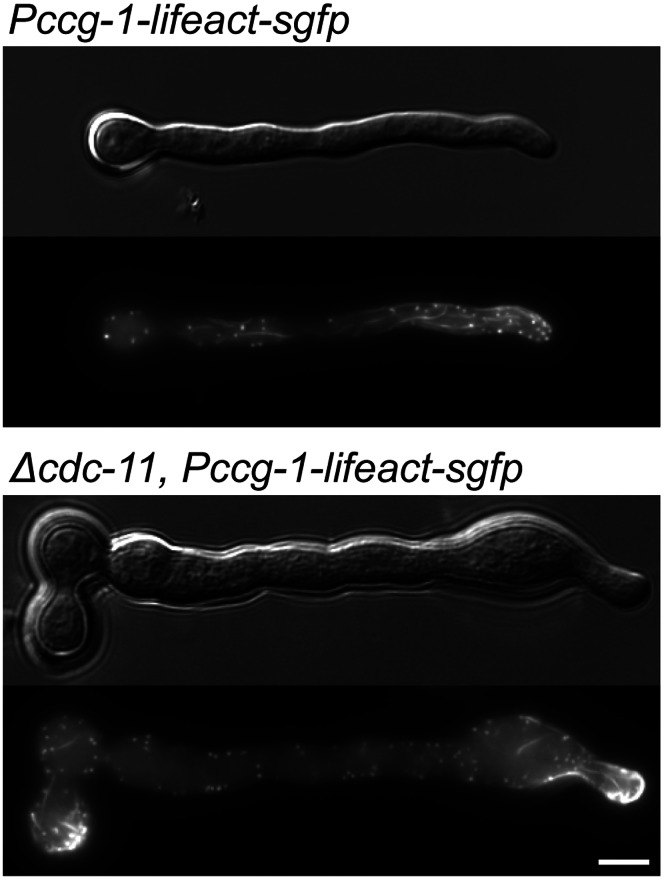
F-actin structures are unaffected in the absence of *cdc-11* **.** Lifeact-GFP-expressing wild-type and *Δcdc-11* strains were incubated in liquid VMM for 3 h then imaged by widefield fluorescence microscopy. F-actin structures appeared as normal although due to the polarity defects of the *cdc-11* null mutant, and actin accumulated at the tip and in the spore body. Scale bar, 5 µm.

To determine the effects of septin deletion on the actin cytoskeleton we generated a *cdc-11* null mutant expressing GFP-Lifeact, which is a marker for F-actin [30], [31]. F-actin structures were unaffected in the absence of septin complexes although due to the polarity defects of the *cdc-11* null mutant, F-actin accumulated at the growing germ tube tip and in the conidium body where new growth had presumably been initiated (Figure 7).

### Septins Form Rings, Fibres, Bar-like Structures and Caps

To visualise the septin cytoskeleton in *N. crassa* we made in-frame fusions of GFP to the 3′ ends of the open reading frames of CDC-3, CDC-10, CDC-11, CDC-12, ASP-1 and ASP-2 so that each resulting fusion protein was expressed from their endogenous promoter as the only copy of the gene. Fluorescent protein (FP)-expressing strains were imaged by wide-field fluorescence microscopy followed by iterative deconvolution. We were unable to visualise CDC-10, ASP-1 and ASP-2 by C-terminal integration of GFP or via overexpression of CDC-10-GFP, ASP-1-GFP and ASP-2-GFP from the *ccg-1* promoter. Overexpression of *cdc-10* and *asp-1* tagged at their N-terminal ends with GFP was successful but we were still unable to visualise ASP-2 even when the expression of the ORF was driven by the strong *ccg-1* promoter. Given that N-terminal overexpression of GFP-CDC-10 and GFP-ASP-1 was efficacious, we generated N-terminal GFP integration cassettes for these genes to ensure that expression level of the fusion construct was as close to wild-type levels as possible. Endogenous expression of GFP-CDC-10 gave an adequate signal-to-noise ratio so this strain was used for further experiments. However, the expression level of the GFP-ASP-1 fusion was too low to allow visualisation so we used the *Pccg1*-s*gfp-asp-1* strain for imaging instead. To determine whether the GFP tag might interfere with septin function, we compared the phenotypes of the septin GFP fusion strains (*cdc-3-gfp*, *gfp-cdc-10*, *cdc-11-gfp*, *cdc-12-gfp*, and *Pccg1-gfp-asp-1*) with those of wild-type and septin-deleted strains. Germination, cell fusion, conidiation, and septation were all similar to wild-type in the septin-GFP strains, indicating that the GFP tags did not interfere with function (Figure S2).

**Figure 8:**
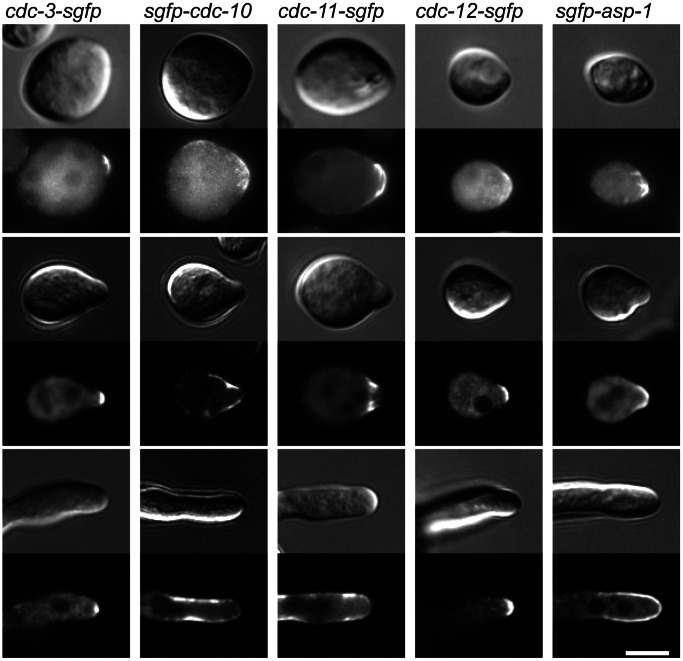
Septins show different patterns of localisation at germ tube tips. Septin-GFP strains were incubated in liquid VMM for 3 h then imaged with DIC and widefield fluorescence microscopy to obtain representative images of germination and development. At the hyphal tip septins localised as a cap (CDC-3-GFP and CDC-12-GFP), an extended cap (GFP-ASP-1) or as a bar-like structures (GFP-CDC-10 and CDC-11-GFP). Scale bar, 5 µm.

All the septins localized at or close to the plasma membrane in germinating conidia at sites of symmetry breaking (Figure 8). Subsequently, at the tips of germ tubes we observed varied patterns of localisation for the different septins (Figure 8). CDC3-GFP and CDC12-GFP were present as a crescent at the growing germ tube tip whereas GFP-CDC10 and CDC11-GFP localised in the form of multiple bar-like structures in the tip region but the signal was noticeably weaker at the tip apex and more intense at the plasma membrane zone approximately 1 µM from the apex. GFP-ASP-1 localised to the plasma membrane but the fluorescent signal was evenly distributed forming a cap that extended ∼5 µM back from the tip. To our knowledge this is the first report of individual septins displaying varied localisation patterns in hyphal tips. Our findings suggest that given their variation in localisation patterns, the individual septins may play different roles in germ tube growth. The localisation pattern was broadly similar for each individual septin throughout the period of germ tube emergence and outgrowth.

**Figure 9:**
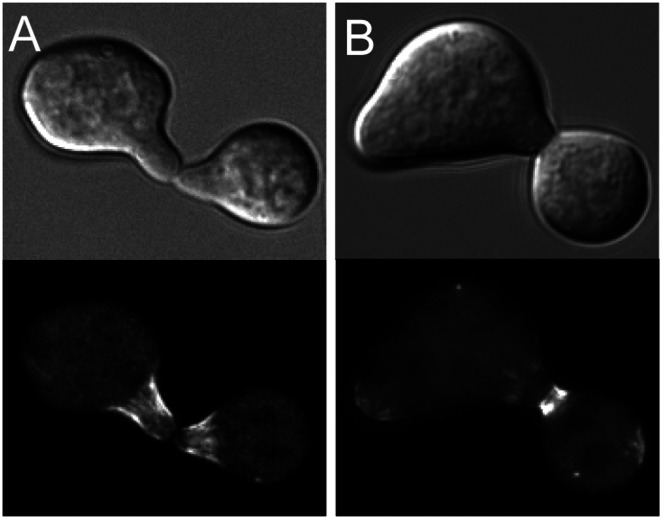
CDC-11 localises to the tips of conidial anastomosis tubes (CATs) and becomes concentrated around the fusion pore. Cells expressing CDC-11-GFP were incubated in liquid VMM for 3 h then imaged with DIC and widefield fluorescence microscopy. (**A**) CDC-11-GFP localised as bar-like structures within the tips of CATs before and after making contact with each other. (**B**) After cell fusion, CDC-11 became concentrated around the fusion pore. Scale bar, 5 µm.

A near identical spatial organisation was observed for septins in CATs before and up to the point of making contact (Figure 9A and data not shown). After fusion, however, we observed that the characteristic bar-like structures for one septin, CDC-11, became concentrated around the fusion pore, indicating a possible role for septins in the terminal stages of cell fusion (Figure 9B). CDC-11-GFP became dispersed in the cytoplasm following fusion (data not shown).

**Figure 10:**
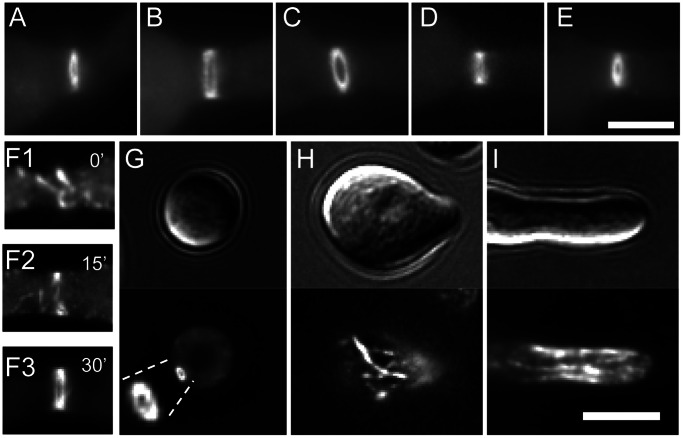
Septins form diverse HO structures. Cells expressing GFP-tagged septins were incubated in liquid VMM for 3 h then imaged with DIC and widefield fluorescence microscopy to obtain representative images of HO structures and septin ring formation. Septin rings formed by CDC-3-GFP (**A**), GFP-CDC-10 (**B**), CDC-11-GFP (**C**), CDC-12-GFP (**D**), and GFP-ASP-1(**E**). (**F1, F2, F3**) The CDC-11-GFP-labelled septin ring was formed by the accumulation of septin fibres. (**G**) Dense septin loops formed by CDC-11-GFP were associated with the cell cortex of an ungerminated conidium. (**H**, **I**) Septin fibres formed by GFP-CDC-10 (**H**) and CDC-11-GFP (**I**) were present in the cytoplasm. Scale bar, 5 µm.

All septins localised as rings at sites of septation suggesting that the heteromeric septin ring complex in *N. crassa* consists of five distinct septins (Figure 10A–E). Ring formation was preceded by an accumulation of septin fibres associated with the cell cortex which eventually concentrated as a ring (Figure 10F). We also observed two other HO septin assemblies in *N. crassa*: loops and fibres (Figures 10G–H).

Dense loops with a diameter of ∼1 uM were visualised with CDC-11-GFP but not with other GFP-tagged septins (Figure 10G). These structures were more commonly observed in ungerminated spores and did not appear to show an association with actively growing regions.

**Figure 11:**
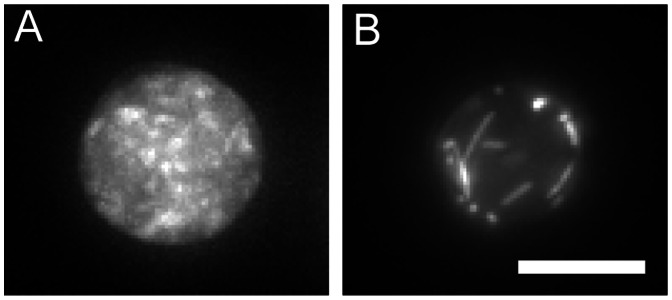
Septins fibres are more abundant when GFP-CDC-10 is overexpressed. Conidia from two GFP-CDC-10 strains were harvested then imaged with fluorescence and DIC microscopy to obtain representative images of septin fibres. When GFP-CDC-10 is expressed from the *Pccg-1* promoter instead of its native promoter then septin fibres are more abundant. The higher fluorescence background in *sgfp-cdc-10* was due to the lower expression of the native *cdc10* promoter compared with the *ccg-1* promoter. Scale bar, 5 µm.

Fibres were approximately 0.2–0.5 µM thick and varied in length from 0.5–5 µM and were found throughout the cytoplasm with some being associated with the cell cortex. Septin fibres were observed with CDC-11-GFP or GFP-CDC-10 but not with other septins, and were more prevalent in ungerminated or slowly growing cells. The number of fibres increased drastically when GFP-CDC-10 was overexpressed by the *ccg-1* promoter compared to endogenous levels (Figure 11). It has been suggested that fibres inhibit new growth [27]; however, we did not observe any inhibition of germination in strain *Pccg-1-sgfp-cdc-10* compared to wild-type or *sgfp-cdc-10* (Figure S2).

To determine if these fibres colocalise with MTs or F-actin, we attempted to generate strains co-expressing GFP-tagged septins and βtub-mCherry or lifeact-tagRFPT. However, the resulting strains exhibited a gross perturbation of cell morphology, inadequate co-expression, and mislocalisation of one or both polymers (data not shown). Similarly, we were unable to generate healthy strains expressing GFP-and RFP-tagged septins (data not shown).

**Figure 12:**
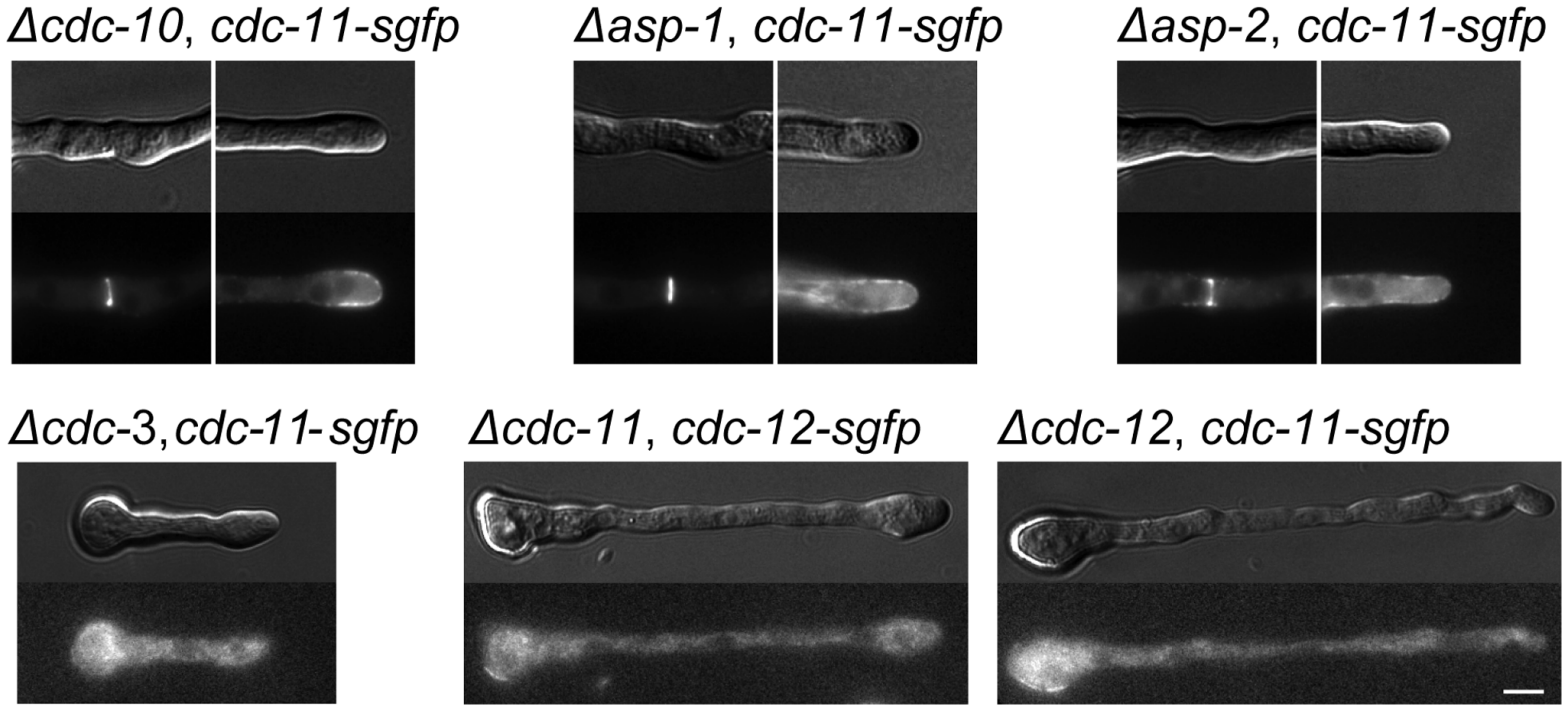
Higher order septin structures fail to form in the absence of CDC-3, CDC-11, or CDC-12. Septin deletion strains expressing CDC-11-GFP or CDC-12-GFP were incubated in liquid VMM for 5 h then imaged with DIC microscopy and widefield fluorescence to obtain representative images of septin ring formation in the absence of a core septin. Scale bar, 5 µm.

To determine if HO septin structures form in the absence of an individual septin we analysed the ability of CDC-11-GFP and CDC-12-GFP to form rings in null mutants. In null mutants lacking *cdc-3*, *cdc-11*, and *cdc-12*, CDC-11-GFP and CDC-12-GFP were only localised to the cytoplasm and septin rings were completely absent (Figure 12). In Δ*cdc-*10, Δ*asp-1* and Δ*asp-2* strains the localisation of CDC-11-GFP rings and tip-associated complexes were indistinguishable from wild-type (compare Figures 6 and 12). These findings suggest that CDC-3, CDC-11, and CDC-12 are essential for oligomerisation and HO structure assembly whereas CDC-10, ASP-1 and ASP-2 are not. Interestingly, although CDC-11-GFP and CDC-12-GFP display different patterns of localisation at germ tube tips, it appears that CDC-11 requires CDC-12 for proper tip distribution and conversely, CDC-12 requires CDC-11 for proper tip distribution (Figure 12).

### Purification and Analysis of Septin Complexes

We utilised tandem affinity purification (TAP) with magnetic beads to detect protein-protein interactions in septin complexes. Single native genomic loci were tagged by the introduction of an integration cassette containing the V5-HAT epitope and hygromycin selection marker. Confirmation of correct integration into the genome by homologous recombination was confirmed by genotyping PCR of transformants. Next, we examined expression of the different epitope-tagged septins. Western blot analysis of whole cell extracts with a monoclonal anti-V5 antibody demonstrated that all of the V5-HAT-tagged septins were expressed with the exception of V5-HAT-ASP-2 (Figure S3). Western analysis demonstrated the presence of two isoforms of CDC-3 and CDC-10 which could represent sumoylated or phosphorylated isoforms of these proteins as has been shown for *S. cerevisiae* Cdc3, Cdc11, and Shs1 [32]–[34].

**Figure 13:**
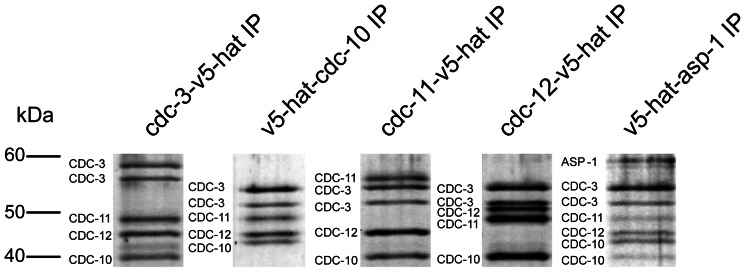
Identification of constituents of septin complexes. SYPRO Ruby-stained SDS PAGE gel of septin complexes. Protein were purified from septin-V5-HAT expressing strains and identified by mass spectrometry. The positions of the molecular mass markers (M, in kDa) are shown at the left of the panel.

Following confirmation of expression, we analysed the respective strains for phenotypic defects. In V5-HAT-expressing strains, germination, fusion, septation, and germ tube emergence was unchanged from wild-type (Figure S2), suggesting that the V5-HAT tag does not interfere with septin function. Immunoprecipitations (IPs) were carried out for the five V5-HAT-labelled septins CDC-3, CDC-10, CDC-11, CDC-12, and ASP-1. Following elution, the purified protein complexes were separated by SDS-PAGE and visualised by coomassie blue staining. Protein bands were excised then identified by tandem mass spectrometry by the SynthSys Centre at the University of Edinburgh. CDC-3, CDC-10, CDC-11, CDC-12 were detected in all five purifications. ASP-1 was only detected when V5-HAT-ASP-1 was used as the IP target. Figure 13 shows a SYPRO ruby-stained gel with protein profiles of the various IPs. No additional proteins were identified as specific components of septin complexes. These results demonstrate that the core septins form an oligomeric complex and suggests that ASP-1, although it probably does not constitute a core subunit of the septin polymer, interacts with it nonetheless.

## Discussion

Septins have been well-studied in budding yeast and are known to have important roles in regulating cytokinesis, cell polarity, morphogenesis and cell wall synthesis [4]. The study of septins in filamentous fungi is still in its infancy but recent reports have revealed new roles for these cytoskeletal polymers. Of particular interest is the finding in filamentous fungi that septins form a variety of HO structures in a single cell and that these structures are regulated by different mechanisms and display different dynamics [7]. In addition, two septins specific to filamentous fungi have been identified in a recent phylogenetic study suggesting that septins may have evolved novel functions in these organisms [2]. To better define the roles of septins in filamentous fungi we studied the six septins present in the *N. crassa* genome using a combination of null mutants, microscopic analysis of GFP tagged alleles, and mass spectrometry of purified complexes.

We found that none of the *N. crassa* septins are essential for cell viability. Similarly, in other filamentous fungi such as *A. nidulans*, *A. gossypii* and *C. albicans* and the fission yeast *S. pombe* none of the septins studied were essential [7], [15], [19], [27], [28], [35]–[37]. This in the stark contrast to the situation in *S. cerevisiae*, where deletion of *cdc3* and *cdc12* was lethal, and *cdc10* and *cdc11* null mutants were viable only at low temperatures [3], [38]. Interestingly, the pathogenic basidomycetes *C. neoformans* and *U. maydis* which both grow by budding in their yeast phases, required core septins for growth at elevated temperatures. Cumulatively these findings highlight a seemingly important link between cell geometry and/or growth mode and the requirement of septins in a particular cell type or species. The phenotypes of *N. crassa* strains lacking the core septins (*cdc-3*, *cdc-10*, *cdc-11*, and *cdc-12*) were indistinguishable with all exhibiting reduced septation, increased germ tube emergence, defective separation of conidia, and swollen and misshapen germ tubes. Although the *Δcdc-10* mutant showed the same phenotype as *cdc-3*, *cdc-11*, and *cdc-12* deletion strains, quantification of multipolarisation, septation, and conidiation defects revealed these were not as greatly perturbed. The *Δasp-1* and *Δasp-2* null mutants did not show any detectable phenotype and behaved as wild-type. Calcofluor white staining of septin mutants showed that despite the misshapen and swollen phenotype of deletion strains, cell walls or septa did not appear to be thickened or abnormal compared to the wild-type suggesting that the phenotypes observed were not due to defective cell wall deposition. This contrasts with *U. maydis* where septin null mutants grown at a restrictive temperature showed a distinctly altered morphology associated with a thickening of the cell wall in the middle of the cell [21]. Similarly, in *C. albicans*, cell wall deposition is perturbed in the absence of *cdc10* or *cdc11* [19]. Taken together these results suggest that *N. crassa*, unlike *U. maydis* and *C. albicans*, does not share the strict requirement for septins in guiding the localisation of the cell wall synthesis machinery.

Given the similarity of the phenotypes for the *cdc-3*, *cdc-10*, *cdc-11*, and *cdc-12* deletion mutants, it is likely that these core septins form a heteromeric complex whose function depends on the presence of these four septins. However, the less severe phenotype observed for the *Δcdc-10* mutant suggests the role played by different septins in the assembly or function of a septin complex varies. Indeed, analysis of double mutants lacking two of the core septins in different combinations revealed an exacerbation of phenotypic defects compared to single deletion mutants, suggesting that some septin function is non-overlapping. It has been shown that in *S. cerevisiae* Cdc10 is phosphorylated by Cla4 and that Cdc10 is thought to link polymeric septin rods together [39], whereas Cdc3 is phosphorylated by the cyclin-dependent kinase Cdc28 and is thought to occupy a different part of the heteroctamer with different binding partners.

The distinct cell polarity defects in *N. crassa* core septins null mutants are reminiscent of the aberrant branching patterns observed in septin deletion strains of *A. nidulans*, and *A. gossypii* [28], [35]. We observed an increased number of germ tubes emerging from conidia and the formation of multiple CATs during cell fusion whereas typically only one CAT is formed per germling in the wild type [40]. These findings suggest that septins influence the maintenance and fidelity of the cell polarity machinery presumably by constraining and corralling this machinery to distinct locations on the plasma membrane, in keeping with the known function of septins functioning as a molecular boundary. The absence of a functional septin complex could feasibly alter the dynamics and/or localisation of important polarity components causing multiple germ tube and/or CAT emergence. Multiple bud emergence has been described in certain *S. cerevisiae* septin mutants [41] and has been ascribed to a failure to successfully corral the polarity machinery.

Although none of the core septins are essential in *N. crassa*, disruption of these genes singly or in combination produces pronounced morphological defects such as misshapen, wider germ tubes that are swollen at the tip. In *C. albicans*, deletion of either of the two non-essential core septins *cdc10* and *cdc11* produced hyphae that appeared as the wild-type but grew with a greater degree of curvature [19]. In *N. crassa*, the morphological defects do not seem to appear to stem from altered deposition of chitin. These morphological changes are therefore likely caused by defects in targeted secretion. Septins are known to interact with the exocyst and polarisome in *S. cerevisiae* [13], and, as such, it is possible that these components are mislocalised or improperly regulated in *N. crassa* in the absence of core septins.

Septation in *N. crassa* initially involves the assembly of a contractile actin ring (CAR) followed by septum formation and subsequent ingrowth, but does not result in complete cytokinesis and cell separation [30], [42], [43]. *Neurospora crassa* core septins are involved in but are not essential for septation – septation is delayed in their absence. The involvement of core septins in septation is unsurprising given their known roles in cytokinesis in other fungi [3], [15]. *Saccharomyces cerevisiae* has a strict requirement for core septins to form septa and complete cytokinesis, whereas in *A. nidulans*, *A. gossypii*, *C. albicans* and *S. pombe* core septin mutants, septa are still formed but in some instances they appear abnormal [19], [28], [35]–[37]. As with other phenotypic behaviour, *N. crassa Δcdc-10* mutants did not display as severe a septation defect as *Δcdc-3*, *Δcdc-11*, and *Δcdc-12* deletion strains again suggesting that it has a slightly different role to other core septins in septin complex formation.

The marked defect in cell separation during conidiation is in sharp contrast to the relatively mild defect in septation in *N. crassa* core septin deletion mutants. The majority of conidia in *N. crassa* are formed by macroconidiation which involves the formation of specialised aerial hyphae (conidiophores) and the subsequent formation of proconidial chains by repeated apical budding followed by sepation between proconidia and their separation into spores [44]. *Neurospora crassa* core septin deletion mutants produce chains of unseparated conidia which suggests that CAR contraction and cell wall/plasma membrane ingrowth fails to occur between proconidia. A role for septins in spore formation has been reported in *A. nidulans* and *C. neoformans* [23], [27], [28]. In these organisms the production of the specialised spore-forming structure, a conidiophore or basidium, respectively, is perturbed rather than the separation of spores. These differences are most likely due to differences in the sequence of events during macroconidium-formation in *N. crassa* compared to *A. nidulans* and *C. neoformans*. In the latter two organisms, septins are probably necessary for the initial cytokinetic events during the morphogenesis of the spore-forming structure and failure to undergo cytokinesis terminates the process. A fraction of cytokinetic events during spore-formation are successful hence the production of separated spores albeit at a reduced rate [23], [28].

We were able to label all of the *N. crassa* septins by integrating GFP at the endogenous locus of the respective genes, with the exception of ASP-2, which we were unable to visualise either by either integration of a knock-in cassette or overexpression. Due to the weak fluorescence of GFP-ASP-1, we used an overexpression fusion construct controlled by the *ccg-1* promoter. In keeping with a previous proposition [45], septin localisation fell into three patterns: localisation to partition as septin rings, localisation to foci of new growth as caps and bar-like structures, and cytosolic localisation as loops and fibres. All septins localised as a ring and were all present at hyphal tips as has been shown for other fungal septins. However, at the tips of germ tubes and CATs we observed three specific patterns of septin localisation: a cap formed by CDC-3 and CDC-12, a band of bar-like structures formed by CDC-10 and CDC-11, and an extended cap formed by ASP-1. These structures/patterns marked the site of germ tube emergence prior to outgrowth and persisted during tip extension. We did not observe the formation of septin rings from these tip-associated complexes as has been found in *A. gossypii*. This is the first report demonstrating the co-existence of different patterns of septin localisation at a hyphal tip. It is possible that the different patterns observed represent different functional roles for septins in maintaining and/or regulating the tip growth apparatus in these hyphae. The presence of septins at the hyphal tip is consistent with their proposed roles as scaffold and boundaries at the plasma membrane. Interestingly, in *C. albicans* the exact pattern of septin localisation at hyphal tips is determined by the cell type; a diffuse band of Cdc10-GFP is found at the tips of germ tubes whereas in pseudohyphae the band is absent and instead Cdc10-GFP is organised into a cap. In this instance different patterns of septin organisation are due to changes in septin regulation during the elaboration of different hyphal types [19].

Another possible role for septins in *N. crassa* comes from their localisation during CAT-mediated cell fusion. During cell fusion, septin localisation at CAT tips is identical to that of germ tubes. Once contact between two CATs is made septins became enriched at the tip apex then gradually rearranged to form the perimeter of the nascent fusion pore, suggesting that they participate in the fusion process. A variety of other proteins are enriched in the tips of CATs growing chemotropically towards each other, remain in CAT tips following cell fusion, and are localized at the fusion pore [46]. These proteins include F-actin [30] which has been shown to interact with septins in mammalian cells (see discussion below).

In addition to rings, caps, and bar-like structures, we observed septins organised into loops and fibres. The dense septins loops, ∼1 µM in diameter, were in ungerminated conidia expressing CDC-11-GFP and a similar structure has been observed in *A. nidulans*, *A. gossypii*, *A. fumigatus* and in mammalian cells treated with anti-actin drugs [10]. In *A. gossypii*, an increase in the number of loops was associated with growth, arrest, heat shock, and treatment with anti-actin drugs led the authors to suggest that these structures are self-assembling reservoirs of septins that form above a certain concentration of free subunits [7]. In *N. crassa*, these loops are not observed in the presence of other HO septin structures or during growth and, as such, it is likely they serve a similar function.

Septin fibres were commonly observed in cells expressing CDC-11-GFP or GFP-CDC-10 but not other septin-GFP fusions. Septin fibres are present in a range of mammalian cells and have been recently found in *A. nidulans*, *A. gossypii*, *A. fumigatus*, *C. neoformans*, *U. maydis*, and chlamydiospores of *C. albicans* [7], [21], [23], [27], [29], [47], [48]. In mammalian, cells septin fibres colocalise with actin stress filaments and/or microtubules and in *C. neoformans* and *U. maydis* partially colocalise with microtubules, although colocalisation of fibres with actin cables was not carried out in these latter two organisms [21], [23]. Our attempts to generate strains co-expressing GFP-tagged septins and βtub-mCherry or Lifeact-tagRFPT were unsuccessful due to the resultant gross perturbation of cell morphology, inadequate co-expression, and mislocalisation.

The septin fibres observed in *N. crassa* appear very similar to those present in *A. nidulans* and *A. fumigatus* [27], [29]; they were 0.2–0.5 µM thick and 0.5–5 µM long, and are associated with the plasma membrane and found in the cytosol. When we overexpressed GFP-CDC-10 we saw a drastic increase in the number of fibres (Figure 11). The accumulation in septin fibres upon overexpression of GFP-CDC-10 suggests that these fibres could serve as storage reservoirs of free GFP-CDC-10 monomers, similar to the proposed role of the septin loops found in quiescent regions/cells [7]. Alternatively, based on the absence of septin fibres in budding yeast and the finding that yeast septin mutants do not produce extra growth foci, it has been proposed that septin fibres may have a role in suppressing new growth [27]. Indeed, the presence of fibres in non-growing regions and cells supports this idea and the notion that fibres are storage reservoirs [27]. However, as overexpression of GFP-CDC-10 in *N. crassa* does not supress germination or germ tube emergence (Figure S2), this implies that fibres are not a growth repressing structure.

To investigate the relationship of the various septins in the assembly of HO structures we analysed the ability of CDC-11-GFP or CDC-12-GFP to form rings and other structures in null mutants. Typically in *S. cerevisiae*, *A. nidulans*, and *A. gossypii* the absence of a single core septin abrogates the formation of HO structures [7], [28], [49], [50] whereas in *U. maydis*, *C. albicans*, and *S. pombe* these structures persist in septin null mutants [19], [21], [51]. We found that in *N. crassa* CDC-3, CDC-11, and CDC-12 are required for ring formation and proper localisation of septin complexes; in their absence the only fluorescence observed was cytoplasmic. Interestingly, although CDC-11-GFP and CDC-12-GFP display different patterns of organisation in the tip growth apparatus, it appears that CDC-11 requires CDC-12 for proper tip localisation and vice versa, CDC-12 requires CDC-11 for proper tip localisation. Conversely, deletion of *cdc-10*, *asp-1*, or *asp-2* did not impede the formation and localisation of septin complexes. From these findings it can be expected that ASP-1 and possibly ASP-2 are “peripheral” septins that could modify or influence septin complexes but are not core constituents. It is possible that ASP-1 performs a similar regulatory role to Shs1 in *S. cerevisiae*, which binds to and interacts with the septin complex. It has been shown that Shs1 is phosphorylated [33] and that it plays a role in regulating the formation and stability of the septin ring complex [8]. Interestingly, *A. nidulans* mutants lacking AspE, the ASP-1 ortholog, showed a marked reduction of septin fibres while other structures are mostly unaffected, suggesting a specific role for this septin in fibre assembly [27]. The ability of *cdc-10* null mutants to form HO structures is consistent with models of septin polymer formation in *S. cerevisiae*, where Cdc10 links septin oligomers together [38]. Similarly, in *U. maydis* the CDC-10 ortholog, Sep4, is not required to form HO assemblies. Septins have been shown to associate with actin in mammalian cells [10], [52]. To determine the effects of septin deletion on the actin cytoskeleton we generated a *cdc-11* null mutant expressing GFP-lifeact, which is a marker for F-actin [30], [31]. F-actin structures were unaffected in the absence of septin complexes although the location of actin cable nucleation was dispersed in germ tube tips compared to the wild-type suggesting that septin complexes help to constrain regions of actin nucleation at the hyphal tip.

We were able to efficiently purify septin complexes from *N. crassa* cell extracts using five of the six septins as the target. The failure to detect expression of V5-HAT-tagged ASP-2 in western blots or in IP experiments, the lack of a phenotype for the *asp-2* null mutant, the inability to express GFP fusions for ASP-2 by knock-in or by overexpression, and the failure to detect ASP-2 in subsequent IP of various septins, indicate that the *asp-2* ORF is not expressed at detectable levels. Septin complexes were isolated in a convenient, rapid dynabead-based method with almost a complete absence of contaminants, confirming that the V5 epitope is highly efficacious for protein purification from *N. crassa*.

The composition of the purified *N. crassa* septin complex is similar to that obtained for *S. cerevisiae* and *C. albicans*; the septin oligomer is composed of CDC-3, CDC-10, CDC-11, and CDC-12 with ASP-1 present as an associating partner but is probably not an integral component of the septin complex.

## Materials and Methods

### Strains and Culture Conditions

*Neurospora crassa* strains used and generated in this study are listed in Table 2. *Neurospora crassa* strains generated during this study were derived from FGSC #2489 (WT *74-OR23-1 A*), FGSC #6103 (*his-3^−^ A*) FGSC #9717 (*his-3^−^ Δmus-51*::*bar^+^ A*), FGSC #9718 (*Δmus-51*::*bar^+^ a*), and FGSC #9719 (*Δmus-52*::*bar^+^ a*) which were obtained from the Fungal Genetics Stock Center (FGSC; School of Biological Sciences, Kansas City, MO). Strains were maintained on Vogel’s minimal medium (VMM) plates with 2% sucrose and 1.5% agar and all manipulations were according to standard *N. crassa* techniques [53]. Hygromycin B (Calbiochem), nourseothricin (Werner BioAgents), and Ignite (phosphinothricin; Sigma-Aldrich) were used at concentrations of 200, 25, and 800 µg/ml, respectively.

**Table 2:** *N. crassa* strains used in this study.

Strain	Genotype	Source
*Wild-type*	*74-OR23-1 A*	FGSC #2489
*his-3^−^*	*his-3 A*	FGSC #6103
*his-3^−^*, *Δmus-51*	*his-3 Δmus-51*::*bar A*	FGSC #9717
*Δmus-51*	*Δmus-51*::*bar a*	FGSC #9718
*Δmus-52*	*Δmus-52*::*bar a*	FGSC #9719
*lifeact-rfp*	*his-3^+^::Ptef-1-lifeact-tagrfpt Δmus-51*::*bar a*	This study
*Δcdc-3*, *Δmus-52*	*Δcdc-3::hph Δmus-52::bar*	This study
*Δcdc-3*	*Δcdc-3::hph*	FGSC #11972
*Δcdc-3*, *Δcdc-10*	*Δcdc-3::hph Δcdc-10::nat*	This study
*Δcdc-3*, *Δcdc-11*	*Δcdc-3::hph Δcdc-11::nat*	This study
*Δcdc-3*, *Δcdc-12*	*Δcdc-3::hph Δcdc-12::nat*	This study
*Δcdc-*3, *cdc-11-sgfp*	*Δcdc-3::hph cdc-11-sgfp::nat*	This study
*cdc-3-sgfp*	*cdc-3-sgfp::hph*	This study
*cdc-3-v5-hat*	*cdc-3-v5-hat::hph*	This study
*Δcdc-10*, *Δmus-51*	*Δcdc-10::hph Δmus-51::bar*	FGSC #11727
*Δcdc-10*	*Δcdc-10::hph*	This study
*Δcdc-10*, *Δcdc-11*	*Δcdc-10::hph Δcdc-11::nat*	This study
*Δcdc-10*, *Δcdc-12*	*Δcdc-10::hph, Δcdc-12::nat*	This study
*Δcdc-10*, *cdc-11-sgfp*	*Δcdc-10::hph cdc-11-sgfp::nat*	This study
*sgfp-cdc-10*	*sgfp-cdc-10::hph*	This study
*Pccg1-sgfp-cdc-10*	*his-3^+^::Pccg-1-sgfp-cdc-10*	This study
*sgfp-cdc-10, bml-mch*	*his-3^+^::Pccg-1-sgfp-cdc-10/Pccg-1-bml mch::nat*	This study
*v5-hat-cdc-10*	*v5-hat-cdc-10::hph*	This study
*Δcdc-11*, *Δmus-51*	*Δcdc-11::hph Δmus-51::bar*	This study
*Δcdc-11*	*Δcdc-11::hph*	FGSC #11971
*Δcdc-11*, *cdc-12-sgfp*	*Δcdc-11::hph cdc-12-sgfp::nat*	This study
*Δcdc-11*, *lifeact-sgfp*	*Δcdc-11::hph his-3^+^::Pccg-1-lifeact-sgfp*	This study
*cdc-11-sgfp*	*cdc-11-sgfp::hph*	This study
*cdc-11-v5-hat*	*cdc-11-v5-hat::hph*	This study
*Δcdc-12, Δmus-51*	*Δcdc-12::hph Δmus-51::bar*	This study
*Δcdc-12*	*Δcdc-12::hph*	This study
*Δcdc-12*, *cdc-11-sgfp*	*Δcdc-12::hph cdc-11-sgfp::nat*	This study
*cdc-12-sgfp*	*cdc-12-sgfp::hph*	This study
*cdc-12-sgfp*, *lifeact-rfp*	*cdc-12-sgfp::hph his-3^+^::Ptef-1-lifeact-tagrfpt*	This study
*cdc-12-v5-hat*	*cdc-12-v5-hat::hph*	This study
*Δasp-1*, *Δmus-51*	*Δasp-1::hph Δmus-51::bar*	This study
*Δasp-1*	*Δasp-1::hph*	This study
*Δasp-1*, *cdc-11-sgfp*	*Δasp-1::hph cdc-11-sgfp::nat*	This study
*Pccg1-sgfp-asp-1*	*his-3^+^::Pccg-1-sgfp-asp-1*	This study
*v5-hat-asp-1*	*v5-hat-asp-1::hph*	This study
*Δasp-2*, *Δmus-51*	*Δasp-2::hph Δmus-51::bar*	This study
*Δasp-2*	*Δasp-2::hph*	This study
*Δasp-2*, *cdc-11-sgfp*	*Δasp-2::hph cdc-11-sgfp::nat*	This study
*v5-hat-asp-2*	*v5-hat-asp-2::hph*	This study

### Transformation and Transformant Selection

Electroporation of *N. crassa* was performed as described previously [54]. Transformants were selected either by growth on selection media containing either hygromycin or nourseothricin or by recovery of histidine prototrophy.

To create single and double gene deletion strains, knock-out cassettes were released from their respective plasmids by digestion with *Not*I and then transformed into strain FGSC #9718 (*Δmus-51::bar*). Genotyping analysis (PCR and Southern blotting) of deletion strains and others was carried out to confirm correct integration of constructs (data not shown). Selected transformants were back-crossed to the wild-type to restore the *mus-51* or *mus-52* gene and provide homokaryotic null mutants. One of the deletion strains obtained from the FGSC (FGSC #11727) was supplied as a heterokaryon and was back-crossed to the wild-type to acquire a homokaryotic deletion strain. Additionally, strain FGSC #11972 was crossed with FGSC #9719 strain to yield strain *Δcdc-3*, *Δmus-52*. FGSC strain #11971 was crossed to FGSC strain #9717 and to NCAB1721 to yield strains *Δcdc-11*, *Δmus-51* and *Δcdc-11*, *lifeact-sgfp*, respectively.

To generate strains expressing epitope-tagged proteins from their native loci, plasmids containing knock-in cassettes were digested with *Not*I and transformed into strain FGSC #9718 (*Δmus-51::bar*) with the exception of *cdc-3* integration cassettes which were digested with *Kpn*I and transformed into strain FGSC #9719 (*Δmus-52::bar*). *cdc-11*-*sgfp::nat* and *cdc-12-sgfp::sgfp* knock-in cassettes were transformed into *Δmus-51* or *Δmus-52* septin null mutants to analyse their localisation in the absence of other septins.

C-terminal and N-terminal overexpression constructs for *cdc-10*, *asp-1*, and *asp-2*, were digested with *Nde*I (or *Ssp*I for pAB222 and pAB322) and targeted to the *his-3* locus of strain FGSC #6103 (*his-3^−^*). To visualize septins simultaneously with microtubules, strain *Pccg1-sgfp-cdc-10* was transformed with undigested pAB46BT to yield strain *sgfp-cdc-10, bml-mch*. An actin/septin co-labelled strain was generated by transforming FGSC #9717 with *NdeI*-digested pAB271 and then transforming the resulting *lifeact-rfp* strain with *NotI*-digested pAB427 (*cdc-11-sgfp* knock-in cassette) to yield strain *cdc-11-sgfp*, *lifeact-rfp*.

FP expression was examined in multiple transformants using a Nikon SMZ1500 stereomicroscope with a GFP (excitation 470/40 nm, 505 nm dichroic mirror, emission 530/40 nm) or RFP filter set (excitation 545/30 nm, 570 nm dichroic mirror, emission 620/60 nm). Intracellular expression patterns were analyzed in at least eight transformants per plasmid construct by widefield fluorescence microscopy. Transformants exhibiting FP expression were selected for subsequent live-cell imaging studies. Expression of V5-HAT-tagged proteins was determined by immunoblotting (see below).

### Engineering of Deletion Cassettes and Epitope Tagging Cassettes

All primers and synthetic oligonucleotides used in this study are listed in Table S1. Plasmids constructed in this study are listed in Table 3. All PCRs were carried out using Phusion polymerase (Finnzymes), with the exception of genotyping PCRs which were carried out using Phire polymerase (Finnzymes). Cycling conditions for both polymerases were as follows: initial denaturation at 98°C for 45 s, followed by 30 cycles of 98°C for 10 s, annealing at 60–70°C (depending on t_m_ of primer set) for 10 s, extension at 72°C for 20 s per kb of amplicon, and then a final extension at 72°C for 60 s. Wild-type genomic DNA was used as a template to amplify genomic sequences.

**Table 3:** Plasmids used in this study.

Plasmid name	Vector backbone	Target/epitiope	Source
pAB415	pRS426	*cdc-3-5f-hph-cdc-3-3f*	This study
pAB425	pRS426	*cdc-3-5f-sgfp-hph-cdc-3-3f*	This study
pAB455	pRS426	*cdc-3-5f-v5hat-hph-cdc-3-3f*	This study
pAB416	pRS426	*cdc-10-5f-hph-cdc-10-3f*	This study
pAB616	pRS426	*cdc-10-5f-nat-cdc-10-3f*	This study
pAB426	pRS426	*cdc-10-5f-sgfp-hph-cdc-10-3f*	This study
pAB226	pCCG::C-Gly::GFP	*his-3-Pccg-1- cdc-10-sgfp*	This study
pAB326	pCCG::N-GFP	*his-3-Pccg-1-sgfp-cdc-10*	This study
pAB526	pRS426	*cdc-105f-hph-Pcdc-10-sgfp-cdc-10ff*	This study
pAB556	pRS426	*cdc-105f-hph-Pcdc-10-v5hat-cdc-10ff*	This study
pAB417	pRS426	*cdc-11-5f-hph-cdc-11-3f*	This study
pAB617	pRS426	*cdc-11-5f-nat-cdc-11-3f*	This study
pAB427	pRS426	*cdc-11-5f-sgfp-hph-cdc-11-3f*	This study
pAB627	pRS426	*cdc-11-5f-sgfp-nat-cdc-11-3f*	This study
pAB647	pRS426	*cdc-11-5f-mch-nat-cdc-11-3f*	This study
pAB457	pRS426	*cdc-11-5f-v5hat-hph-cdc-11-3f*	This study
pAB418	pRS426	*cdc-12-5f-hph-cdc-12-3f*	This study
pAB618	pRS426	*cdc-12-5f-nat-cdc-12-3f*	This study
pAB428	pRS426	*cdc-12-5f-sgfp-hph-cdc-12-3f*	This study
pAB628	pRS426	*cdc-12-5f-sgfp-nat-cdc-12-3f*	This study
pAB458	pRS426	*cdc-12-5f-v5hat-hph-cdc-12-3f*	This study
pAB419	pRS426	*asp-1-5f-hph-asp-1-3f*	This study
pAB429	pRS426	*asp-1-5f-sgfp-hph-asp-1-3f*	This study
pAB229	pCCG::C-Gly::GFP	*his-3-Pccg-1-asp-1-sgfp*	This study
pAB329	pCCG::N-GFP	*his-3-Pccg-1-sgfp-asp-1*	This study
pAB529	pRS426	*asp-1-5f-hph-Pasp-1-sgfp-asp-1-3f*	This study
pAB559	pRS426	*asp-1-5f-hph-Pasp-1-v5hat-asp-1-3f*	This study
pAB412	pRS426	*asp-2-5f-hph-asp-2-3f*	This study
pAB422	pRS426	*asp-2-5f-sgfp-hph-asp-2-3f*	This study
pAB222	pCCG::C-Gly::GFP	*his-3-Pccg-1- asp-2-sgfp*	This study
pAB322	pCCG::N-GFP	*his-3-Pccg-1-sgfp-asp-2*	This study
pAB552	pRS426	*asp-2-5f-hph-Pasp-2-v5hat-asp-2-3f*	This study
pAB84BT	pRS426	*Pccg-1-bml-mch-nat*	This study

We used yeast recombinational cloning (YRC) to generate deletion cassettes (“knock-out”) and epitope-tagging cassettes (“knock-in”) [55], [56]. Three epitopes, GFP, mCherry and V5-HAT, were used to modify genes at their native loci. GFP and mCherry serve as markers for protein localisation whereas the V5-HAT epitope consists of two distinct epitopes (V5 is a viral epitope and HAT [histidine affinity tag] is a natural 19-aa poly histidine tag derived from chicken lactate dehydrogenase) and was utilised for tandem affinity purification of septin complexes. DNA fragments consisting of targeting flanks, a selection marker and, for knock-in constructs, an epitope were co-transformed into the yeast strain FY834 [57], along with the yeast shuttle vector pRS426 [58] digested with *Xho*I and *Eco*RI, for assembly in yeast via its endogenous recombination system [55], [56]. Yeast transformations were carried out with the lithium acetate/PEG method [59]. After 3 days of growth at 30°C, yeast colonies were harvested and pooled, then plasmid DNA recovered and resuspended in 20 µL of 10 mM Tris-Cl pH 8.0, as described previously [60]. Two microliters of the DNA suspension was electroporated into *Escherichia coli* DH5α. Following restriction digest screening and confirmation of the correct vectors by DNA sequencing, the cassette was cut out with the appropriate restriction enzymes and transformed into *Δmus-*51 or *Δmus-*52 *N. crassa* strains.

The final vectors consisted of targeting flanks specific for the gene of interest and functional elements (henceforth referred to as modules) such as dominant selection markers and epitopes (for integration cassettes). The procedure was simplified by generating standardised modules via PCR which contain defined regions of homology at the 5′ and 3′ end of the DNA fragment. This allows a variety of knock-out and knock-in cassettes to be created with the same targeting flanks by swapping in or out different modules (*i.e.* substituting the *hph* selection module for the *nat* selection module or substituting the GFP module for the V5-HAT module).

To generate targeting flanks, three ∼1 kb regions were amplified from the genes of interest (*cdc-3*, *cdc-10*, *cdc-11*, *cdc-12*, *asp-1*, and *asp-2*). Approximately ∼1 kb upstream of the gene of interest was amplified using primers P1 and P2 to yield flank A. Flank B consists of ∼1 kb of the region upstream of the gene’s stop codon and was amplified using primers P3 and P4. Flank C contains ∼1 kb of the UTR region downstream of the gene’s stop codon and was amplified using primers P5 and P6. Flanks A and C together with a marker module were used to create deletion cassettes whereas flanks B and C, in addition to a marker module and epitope module, were used to create C-terminal epitope tagging cassettes. For three genes (*cdc-10*, *asp-1*, and *asp-2*) C-terminal tagging by knock-in or overexpression was unsuccessful; we therefore created N-terminal knock-in cassettes. To generate N-terminal knock-in cassettes different targeting flanks were required. Flank D contains ∼1 kb of the region between base −2500 and −1500 upstream of the genes start codon and was amplified using primers N1 and N2. Approximately ∼1 kb directly upstream of the gene of interest was amplified using primers N3 and N4 to yield flank E. Flank F contains ∼1 kb of the region downstream of the gene’s start codon and was amplified using primers N5 and N6. N-terminal knock-in cassettes were constructed with these three flanks (D, E, and F), a marker module and an epitope module. All targeting flanks were amplified with a 30 bp extension which corresponds to a region of homology at the 5′- and 3′-end of the various modules or the linearised pRS426.

The two dominant selection markers used in YRC, the *hph* gene and *nat* gene, confer hygromycin resistance and nourseothricin resistance in *N. crassa*, respectively. The *hph* module was amplified from pGFP::hph::loxP [61] with the primers HPH YRC CC Fw and HPH YRC DD Rv. The *nat* module was amplified from pD-Nat [62] with the primers NAT YRC CC Fw and NAT YRC DD Rv. The GFP module for C-terminal tagging was amplified from pGFP::hph::loxP with the primers GFP YRC BB Fw and GFP YRC CC Rv. For N-terminal tagging the GFP module was amplified from pGFP::hph::loxP with the primers GFP YRC NT Fw and GFP YRC NT BB Rv. The mCherry module for C-terminal tagging was amplified from pRset-mCherry [63] with the primers MCH YRC BB Fw and MCH YRC CC Rv. The V5-HAT epitope was constructed *de novo* with *N. crassa*-codon optimized oligonucleotides V5HAT YRC A, V5HAT YRC B, and V5HAT YRC C which are partially overlapping and contain a 10× glycine linker at the 5′-end, the V5 and HAT sequences joined by a 5× glycine-alanine linker, and a region of defined homology at the 3′-end. After boiling for 5 min, the oligos were incubated at room temperature for 30 min to anneal them, then co-transformed into yeast along with *cdc-3* targeting flanks B and C, the hph module and pRS426 linearised with *Xho*I and *Eco*RI. The resulting plasmid, pAB455, was checked by DNA sequencing and then used as a PCR template to generate the V5-HAT module for C-terminal tagging with primers V5HAT YRC BB Fw and V5HAT YRC CC Rv. For N-terminal tagging the V5-HAT module was amplified from pAB455 with primers V5HAT YRC NT Fw and V5HAT YRC NT BB Rv.

As the C-terminal GFP knock-in cassettes failed to provide any detectable signal for *cdc-10*, *asp-1*, and *asp-2* (data not shown), we constructed C-terminal and N-terminal overexpression constructs using the In-Fusion® PCR cloning kit (Clontech). For C-terminal overexpression constructs, the *cdc-10*, *asp-1*, and *asp-2* genes were amplified, without the stop codon, with the primers CDC10 IF Fw and CDC10 P4 Rv, ASP 1 IF Fw and ASP 1 P4, or ASP 2 IF Fw and ASP 2 P4, respectively. The PCR products were purified using a DNA clean and concentrator kit (Zymo Research) then integrated into *Xba*I- and *Pac*I-digested pCCG::C-Gly::GFP [61] using the In-Fusion PCR cloning kit to give plasmids pAB226 (*Pccg-1-cdc-10-sgfp*), pAB229 (*Pccg-1-asp-1-sgfp*), and pAB222 (*Pccg-1-asp-2-sgfp*). Similarly, for N-terminal overexpression constructs, the *cdc-10*, *asp-1*, and *asp-2* genes were amplified with the primers CDC10 IF NT Fw and CDC10 IF NT Rv, ASP 1 IF NT Fw and ASP 1 IF NT Rv, and ASP 2 IF NT Fw and ASP 2 IF NT Rv, respectively. The purified PCR products were then integrated into *Xba*I- and *Asc*I-digested pCCG::N-GFP [61] using the In-Fusion PCR cloning kit to yield plasmids pAB326 (*Pccg-1-sgfp-cdc-10*), pAB329 (*Pccg-1-sgfp-asp-1*), and pAB322 (*Pccg-1-sgfp-asp-2*).

To visualise septins simultaneously with actin or microtubules we created a lifeact-tagRFP-T and a tubulin-mCherry fusion construct. The lifeact-tagRFP-T fusion was made from the plasmid pAB261 which contains Lifeact-GFP under the control of the *tef-1* promoter [30]. The tagRFP-T ORF was amplified from pAL5 with the primers TAGRFPT IF Fw and TAGRFPT IF Rv. GFP was excised from pAB261 by digestion with *Pac*I and *Eco*RI and the linearised plasmid was gel-extracted. The tagRFP-T PCR product was purified using a DNA clean and concentrator kit (Zymo Research) then integrated into *Pac*I- and *Eco*RI-digested pAB261 with the In-Fusion® PCR cloning kit to yield plasmid pAB271 (*Ptef-1-lifeact-tagrfpt*). The tubulin-mCherry fusion was made with YRC. The 2647 bp *Pccg-1-bml* sequence was amplified from plasmid pMF309 [64] with the primers BTUB YRC Fw, which contains 30 bp of homology at the 3′-end of the linearised pRS426, and BTUB YRC Rv, which contains 30 bp of homology at the 5′-end of the mCherry module. The *Pccg-1-bml* sequence was co-transformed into yeast along with the mCherry module, the linearised pRS426 shuttle vector and a *nat* selection module modified to include a 30 bp region of homology to the 5′-end of the linearised pRS426. The resulting plasmid, pAB46BT, was isolated from yeast and screened as described above. DNA sequencing was carried out on all vectors to confirm in-frame cloning of the fusion constructs and correct amplification by PCR.

### Protein Extraction, Affinity Purification and Immunoblotting

Antibody-conjugated magnetic beads were used for protein purification. To purify protein complexes we used anti-V5 antibody conjugated Dynabeads and charged His tag isolation (HTI) Dynabeads. We used two epitopes to label the *N. crassa* septin complexes; a V5 epitope, derived from the V5 simian virus and a poly-histidine sequence from chicken lactate dehydrogenase (HAT), which is known to be a superior alternative to the commonly used 6xHis tag [65]. The double epitope tag was created *de novo* using three overlapping synthetic oligonucleotides that were combined using YRC. We compared the efficacy of one-step and two-step affinity purification in immunoprecipitating CDC-12 and also differences in the eluted protein profiles using two buffers: 0.1 M glycine-HCl (pH 2.5) and hot (70°C) SDS sample loading buffer. The profiles of proteins isolated using the one-step purification with anti-V5 beads were identical to that obtained with the two-step method (Figure S4). In both cases negligible non-specific interactions were detected in untagged wild-type whole cell extract controls using anti-V5 beads, whereas HTI bead purification of whole cell extracts from wild-type and CDC-12-V5-HAT gave similar profiles. The HAT tag is functional, as demonstrated by the subsequent pulldown of the CDC-12-V5-HAT complex by anti-V5 beads. However, the large degree of non-specific contaminants bound to the HTI beads and the efficacy of the one-step anti-V5 bead IP, allowed us to exclude this step from the purification method.

To determine if the protein complex was completely eluted from the beads and to test for the elution of non-specific interactants from the beads, we monitored the protein profiles of immunoprecipitates eluted in 0.1 M glycine-HCl (pH 2.5) and hot (70°C) SDS sample loading buffer. Elution in glycine-HCl allows reuse of the beads but may leave proteins bound to the beads whereas elution in hot SDS sample loading buffer completely strips beads of protein but precludes reuse of the beads. A comparison of elution profiles is shown in Figure S4. The elution profiles for both buffers are essentially identical demonstrating that all bound proteins are eluted in both cases. However, an additional 25 kDa interactant is present in the SDS eluate in both the wild-type and *cdc-12-v5-hat* strains, which suggests that this is a non-specific interactant and binds to the bead surface rather than to the bead-bound complex. This contaminant was absent from the glycine-HCl eluate, and therefore all further elutions were carried out using 0.1 M glycine-HCl (pH 2.5).

All protein purification steps were performed at 4°C. *Neurospora crassa* strains expressing V5-HAT-tagged proteins were grown at 35°C for 2 days in 2 l flasks containing 500 ml of liquid VMM. The cultures were harvested by filtration through Mira-cloth (Calbiochem), washed twice with 250 ml ddH_2_O, and pressed between paper towels to remove excess water. The collected mycelium (∼10 g) was then frozen in liquid nitrogen and ground into a fine powder in a pre-cooled pestle and mortar. Ice-cold HG extraction buffer (50 mM HEPES [pH7.4], 137 mM NaCl, 10% glycerol freshly supplemented with a protease inhibitor cocktail [complete, EDTA-free tablet, Roche] and 1 mM PMSF) was added to the frozen grindate (5 ml of buffer/g of grindate) which was resuspended by vortexing for three 20 s pulses, inverted once every minute for 5 min and then centrifuged for 2370×g at 4°C for 10 min. The supernatant was carefully removed and used immediately for affinity purification. For affinity purification, two types of Dynabeads were used: His tag isolation (HTI) Dynabeads (Invitrogen) and anti-V5 conjugated Dynabeads (Invitrogen). Anti-V5 monoclonal antibody (Invitrogen) was coupled to M-270 epoxy Dynabeads (Invitrogen) at a concentration of 10 mg/ml according to the manufacturer’s instructions. Both types of Dynabeads were washed three times in HG extraction buffer before adding to supernatants. For tandem affinity purification 100 µl of HTI Dynabeads was added to 10 ml of supernatant and incubated on a roller at 4°C for 10 min. HTI Dynabeads were collected with a Dynal magnet (Invitrogen), washed three times in HTI wash buffer (50 mM HEPES [pH7.4], 300 mM NaCl, 0.01% Tween-20) and the complex eluted from the beads with two washes of 100 µl HTI elution buffer (50 mM HEPES [pH7.4], 150 mM NaCl, 300 mM imidazole, 0.01% Tween-20). The eluate was added to 100 µl anti-V5 Dynabeads and incubated on a roller at 4°C for 1 h. Anti-V5 Dynabeads Beads were collected with a Dynal magnet and washed twice in antibody Dynabead wash buffer (ADWB; 50 mM HEPES pH7.4, 150 mM NaCl, 0.02% Tween-20), once in ADWB minus Tween-20 and the proteins eluted twice in 20 µl 100 mM glycine-HCl (pH 2.5). For single step affinity purifications with anti-V5 Dynabeads, 250 µl anti-V5 Dynabeads were added to 4 ml of cell extract. The remaining purification steps were carried out exactly as described for the immunoprecipitation stage of the tandem purification. Purified protein samples were mixed with one-quarter volume of 4× sample buffer (0.2 M Tris-HCl, pH 6.8, 8% (w/v) SDS, 40% glycerol, 10% β-mercaptoethanol, 0.025% bromophenol blue) for SDS–PAGE. Samples were boiled for 5 min, and then separated by 10% SDS–PAGE. Gels were stained with either SYPRO Ruby (Invitrogen) or Safe blue (Invitrogen). For immunoblots, equal amounts of protein (100 µg) were separated by 10% SDS-PAGE, transferred to an Amersham Hybond nylon membrane in CAPS transfer buffer (10 mM CAPS [pH11], 10% methanol) for 80 min at 80 V and the membrane was then blocked in PBS, 2% skimmed milk and 0.2% Tween-20. V5-HAT-tagged proteins were probed using mouse monoclonal anti-V5 antibody (Invitrogen; diluted to 1∶10,000) and goat anti-mouse IgG- IRDye 800CW conjugate (LiCOR; diluted to 1∶20,000) in PBS, 2% skimmed milk, 0.02% Tween-20. Membranes were washed for 30 min in PBS then imaged using a LiCOR Odyssey scanner.

### Mass Spectrometry

Destained SDS-PAGE gel bands were digested with sequencing grade modified porcine trypsin (Promega, ∼5–10 ng/µl) solutions in 50 mM ammonium bicarbonate, and evaporated to dryness by vacuum centrifugation. Micro-HPLC/MS/MS analyses were performed using an online system consisting of a micro-pump Agilent 1200 binary HPLC system (Agilent) coupled to a hybrid LTQ Orbitrap XL instrument (Thermo-Fisher). The LTQ was controlled through Xcalibur 2.0.7 and LTQ Orbitrap XL MS2.4SPI. Capillary Picotip columns (10 cm long, 360 mm outer diameter, 75 mm inner diameter) with a 15 mm tip opening and fitted with a borosilicate frit were obtained from New Objective (Presearch). Fused-silica tubing was from Composite Metal. Samples were analysed on a 2 h gradient for data dependent analysis. MS/MS data were searched using Mascot version 2.2 (Matrix Science Ltd.), against the Neurospora genome and annotations database (http://www.broad.mit.edu/annotation/fungi, accessed January 2012).

### Live-cell Imaging

Conidia were collected from 3- to 5-day-old cultures and suspended in VMM. In all experiments, unless stated otherwise, cells were incubated in Lab-Tek 8-well chamber-slides (Nalge-Nunc International) for 3–5 h in 250 µl of VMM at a concentration of 10^6^ cells/ml then imaged. Imaging was carried out at room temperature. Images were collected using a Nikon TE2000 microscope with: a Nikon100×/1.4 NA Plan-Apo objective; CoolLED PE-2 illuminator with LED excitation centred at 380 nm, 470 nm, and 550 nm; Chroma filter sets (for GFP: excitation 470/40 nm, 495 nm LP dichroic mirror, emission 525/50 nm; for RFP: excitation 535/50 nm, 565 nm LP dichroic mirror, emission 590 nm LP; for calcofluor white: excitation 355/50 nm, 400 nm LP dichroic mirror, emission 420 nm LP); Hammamatsu Orca-ER CCD camera; and MetaMorph software (Universal Imaging) for image acquisition. Exposure times ranged from 100–300 ms. To acquire 3D (x,y, and z) images, 10–15 widefield fluorescence images were captured at 0.4 µm steps using 2×2 camera binning. These images were processed through 10 iterative deconvolutions using AutoQuant image analysis software (Media Cybernetics). Projections and further processing steps were carried out with ImageJ software (rsbweb.nih.gov/ij/). To stain cell walls, cells were labelled for 30 min with 0.1 µg/ml calcofluor white (prepared from 1 mg/ml stock in ethanol; Sigma).

### Quantification of Conidium Germination, Cell Fusion and Septation

Conidial germination was quantified as the percentage of conidia possessing one or more protrusions/hyphae whilst cell fusion was quantified as the percentage of conidia or conidial germlings involved in fusion. Cell fusion during colony initiation involves the fusion of specialized cell protrusions termed conidial anastomosis tubes (CATs) [40], [46]. The number of unseparated conidia was quantified as the percentage of cells attached to another cell at time 0 h. Septation was quantified as the percentage of cells with a septum and cells were scored as having multiple germ tubes if more than one germ tube had emerged from conidia. Statistical analysis (t-test) was performed using ‘R’ (http://www.r-project.org/).

## Supporting Information

Figure S1 **Cell wall deposition is unaffected in septin deletion strains.** Wild-type and septin deletion strains were incubated in liquid VMM for up to 5 h, stained with calcofluor white then imaged with fluorescence microscopy. Scale bar, 5 µm.
(PDF)

Figure S2 **Germination, cell fusion, septation, germ tube emergence and conidia formation in the septin-GFP or septin-V5-HAT strains are unaffected compared to wild-type.** (**A)** Strains were incubated in liquid VMM for 3 h then counted for germination and cell fusion (*n* = 300). (**B**) The number of unseparated cells in various strains was counted before incubation, the number of cells with more than two germ tubes was counted following 3 h of incubation in liquid and the number of septate cells was determined with DIC microscopy after 5 h of incubation (*n* = 300). Differences in the assessed parameters between transformant strains and wild-type were statistically not significant (all p>0.1).
(PDF)

Figure S3 **Western blot analysis of septin-V5-HAT-expressing strains.** Whole-cell extracts from various strains (in italics) were separated by SDS-PAGE then probed with αV5. The positions of the molecular mass markers (M, in kDa) are shown at the left of the panel.
(PDF)

Figure S4 **Comparison of one- and two-step purification and elution buffers.** (**A**) Comparison of one- and two-step purification of whole-cell extracts from wild-type and *cdc-12-v5-hat* strains with elution in 0.1 M glycine-HCl (pH 2.5). Lanes 1 and 5, whole cell extract. Lanes 2 and 6, first step of two-step purification using HTI beads. Lanes 3 and 7, second step of two-step purification using αV5-beads. Lanes 4 and 8, one-step purification using αV5-beads. (**B**) Comparison of one- and two-step purification of whole-cell extracts from wild-type and *cdc-12-v5-hat* strains with elution in hot SDS buffer. Lanes 1 and 5, whole cell extract. Lanes 2 and 6, first step of two-step purification using HTI beads. Lanes 3 and 7, second step of two-step purification using αV5-beads. Lanes 4 and 8, one-step purification using αV5-beads. The positions of the molecular mass markers (in kDa) are shown at the left of the panel.
(PDF)

Table S1 **Oligonucleotide primers used in this study.**
(PDF)
