# Supplementary figures and images for: Septins Are Important for Cell Polarity, Septation and Asexual Spore Formation in Neurospora crassa and Show Different Patterns of Localisation at Germ Tube Tips
Source: PLoS One. 2013 May 14;8(5):e63843. doi: 10.1371/journal.pone.0063843 (PMC3653863; doi:10.1371/journal.pone.0063843)

*WT*

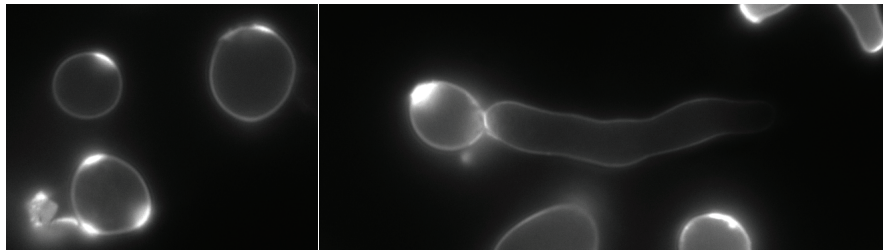

*Δcdc-3*

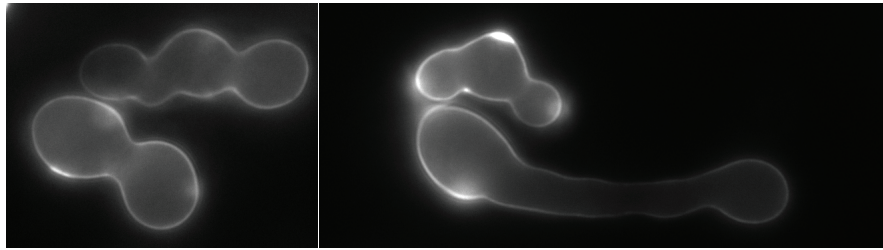

*Δcdc-10*

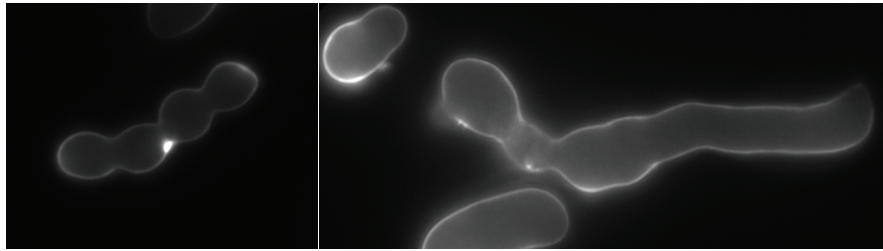

*Δcdc-11*

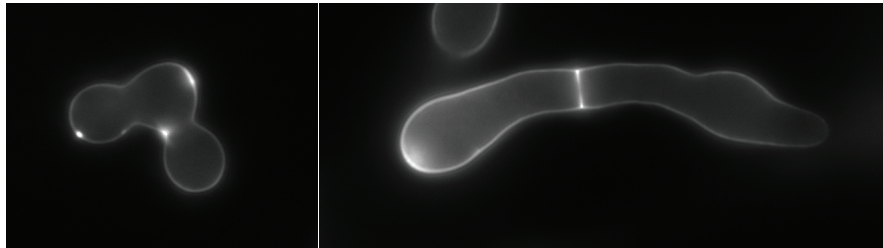

*Δcdc-12*

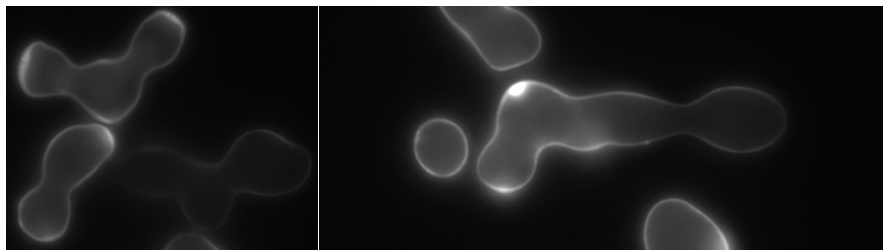

*Δasp-1*

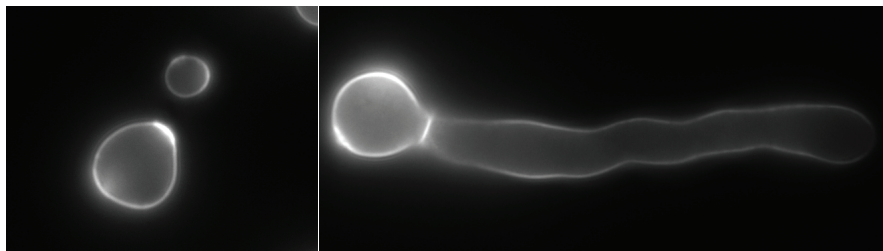

*Δasp-2*

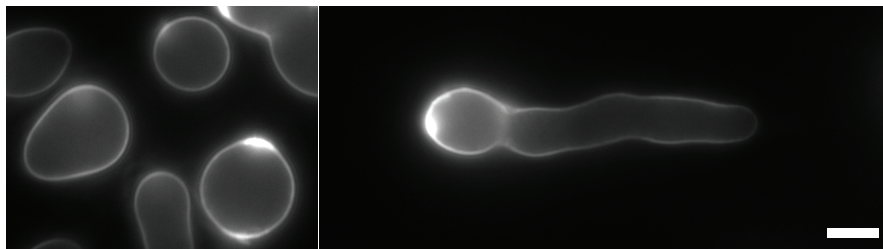

Supplement: Figure S1 — Cell wall deposition is unaffected in septin deletion strains. Wild-type and septin deletion strains were incubated in liquid VMM for up to 5 h, stained with calcofluor white then imaged with fluorescence microscopy. Scale bar, 5 µm. (PDF) [file pone.0063843.s001.pdf]

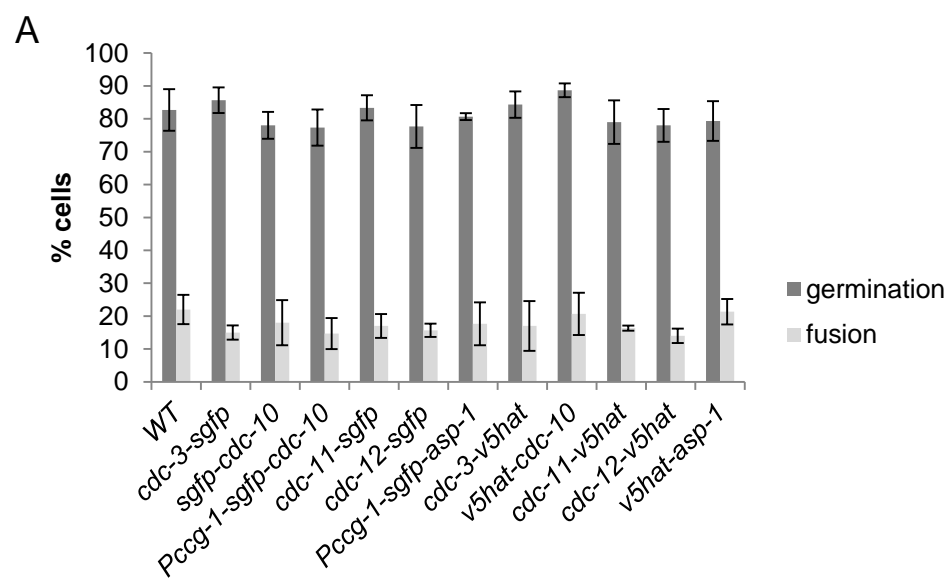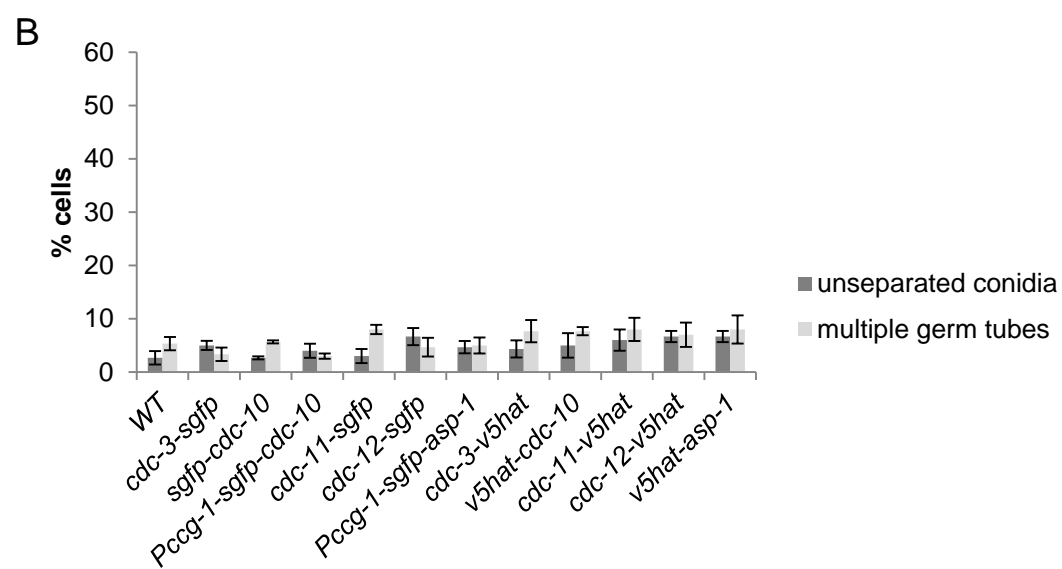

Supplement: Figure S2 — Germination, cell fusion, septation, germ tube emergence and conidia formation in the septin-GFP or septin-V5-HAT strains are unaffected compared to wild-type. (A) Strains were incubated in liquid VMM for 3 h then counted for germination and cell fusion (n = 300). (B) The number of unseparated cells in various strains was counted before incubation, the number of cells with more than two germ tubes was counted following 3 h of incubation in liquid and the number of septate cells was determined with DIC microscopy after 5 h of incubation (n = 300). Differences in the assessed parameters between transformant strains and wild-type were statistically not significant (all p>0.1). (PDF) [file pone.0063843.s002.pdf]

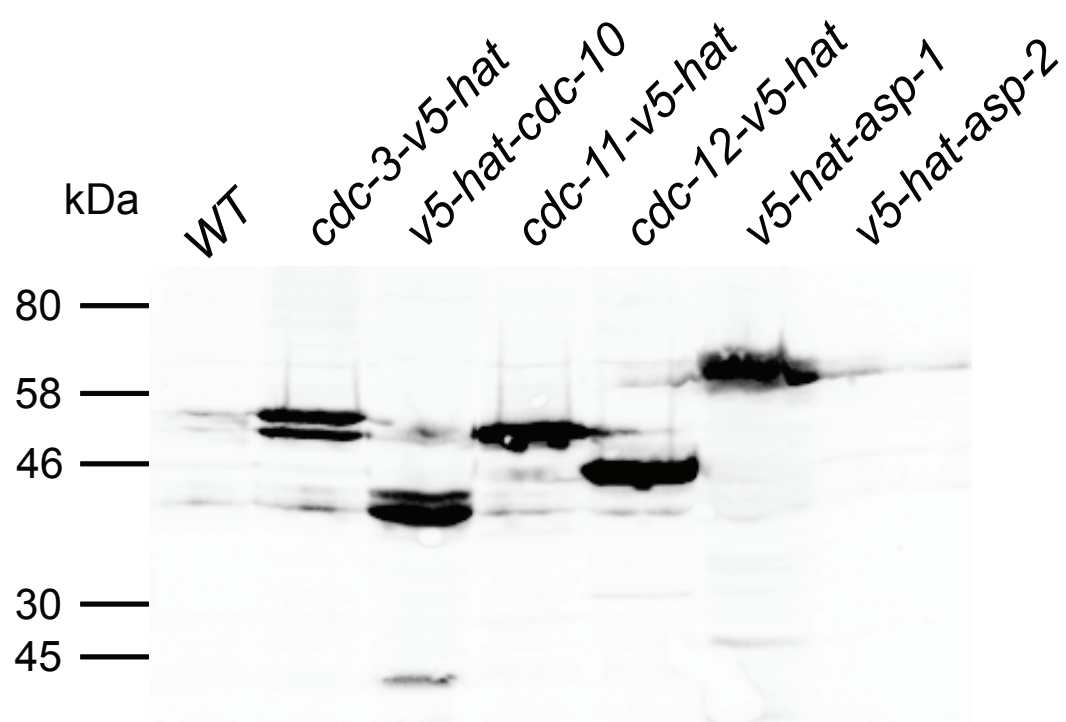

Supplement: Figure S3 — Western blot analysis of septin-V5-HAT-expressing strains. Whole-cell extracts from various strains (in italics) were separated by SDS-PAGE then probed with αV5. The positions of the molecular mass markers (M, in kDa) are shown at the left of the panel. (PDF) [file pone.0063843.s003.pdf]

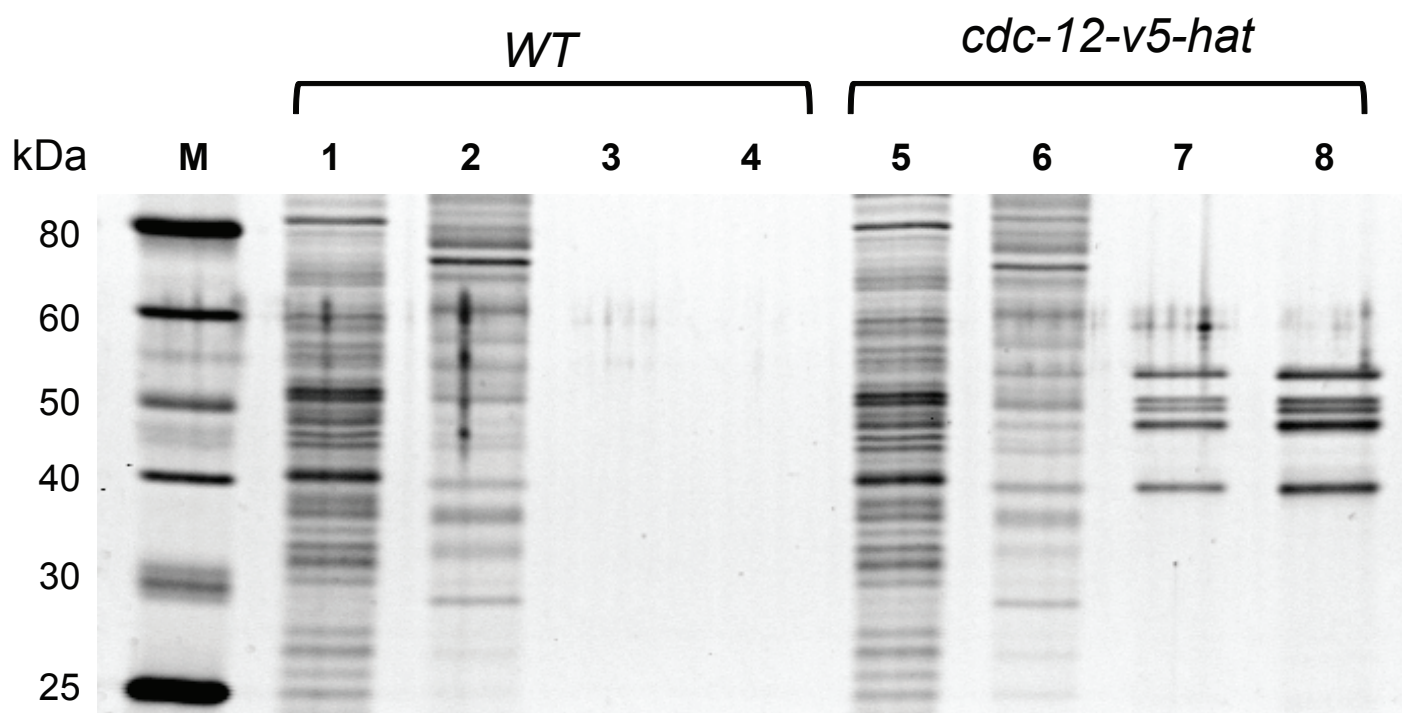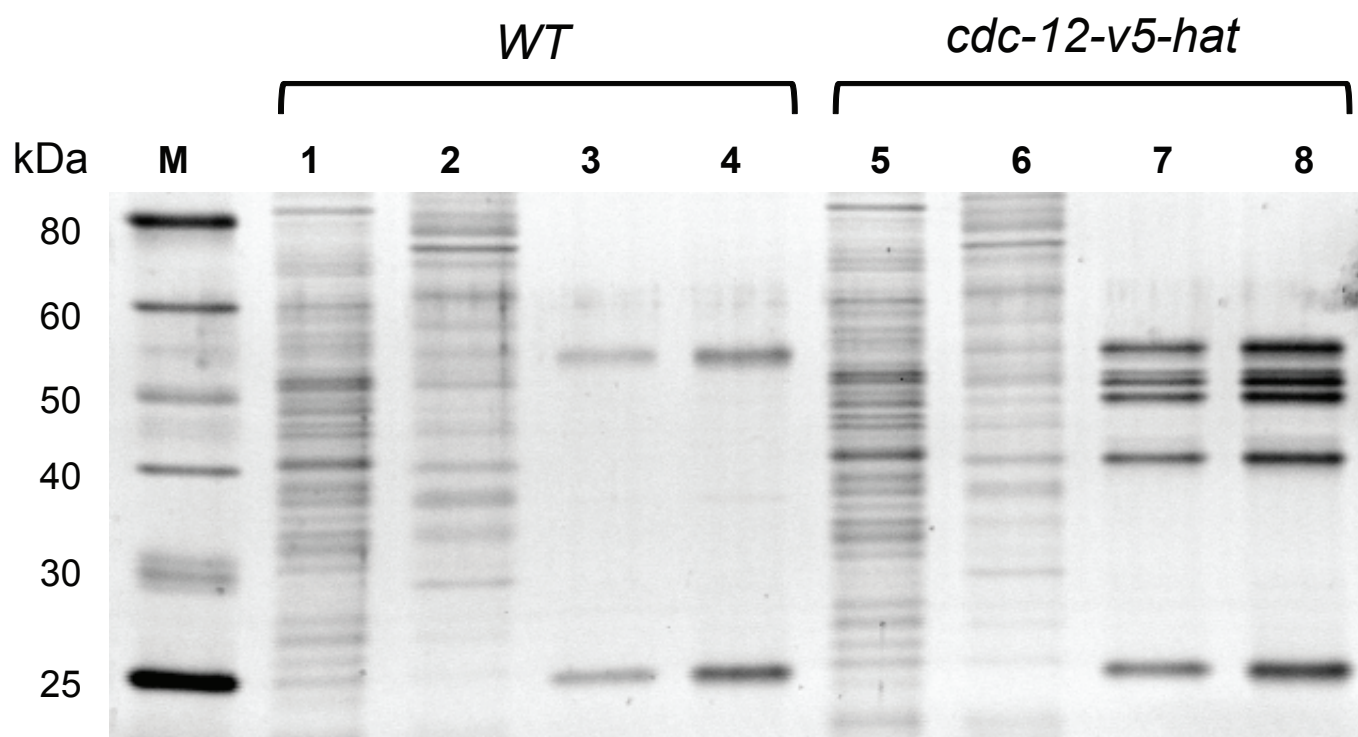

Supplement: Figure S4 — Comparison of one- and two-step purification and elution buffers. (A) Comparison of one- and two-step purification of whole-cell extracts from wild-type and cdc-12-v5-hat strains with elution in 0.1 M glycine-HCl (pH 2.5). Lanes 1 and 5, whole cell extract. Lanes 2 and 6, first step of two-step purification using HTI beads. Lanes 3 and 7, second step of two-step purification using αV5-beads. Lanes 4 and 8, one-step purification using αV5-beads. (B) Comparison of one- and two-step purification of whole-cell extracts from wild-type and cdc-12-v5-hat strains with elution in hot SDS buffer. Lanes 1 and 5, whole cell extract. Lanes 2 and 6, first step of two-step purification using HTI beads. Lanes 3 and 7, second step of two-step purification using αV5-beads. Lanes 4 and 8, one-step purification using αV5-beads. The positions of the molecular mass markers (in kDa) are shown at the left of the panel. (PDF) [file pone.0063843.s004.pdf]
