# Supplementary material for: Septins Are Important for Cell Polarity, Septation and Asexual Spore Formation in Neurospora crassa and Show Different Patterns of Localisation at Germ Tube Tips
Source: PLoS One. 2013 May 14;8(5):e63843. doi: 10.1371/journal.pone.0063843 (PMC3653863; doi:10.1371/journal.pone.0063843)
Supplement: Table S1 — Oligonucleotide primers used in this study. (PDF) [file pone.0063843.s005.pdf]

Table S1. Oligonucleotide primers used in this study

**Primer name**	**Sequence**
CDC-3 P1	GTAATACGACTCACTATAGGGCGAATTGGGTACCacccgtcaggccagtggaca
CDC-3 P2	CGATAAGCTTGATATCGAATTCTTACTTGTAtgcacccggcatgtgtcttc
CDC-3 P3	GTAATACGACTCACTATAGGGCGAATTGGGTACCcttctaccttgagcgacaacc
CDC-3 P4	GCCTCCGCCTCCGCCTCCGCCGCCTCCGCCtcggagagaaaatcccttcc
CDC-3 P5	CAAAGGAATAGAGTAGATGCCGACCGGGATAACcatgttatcccgcatacacc
CDC-3 P6	CTCACTAAAGGGAACAAAAGCTGGAGCTCCtgagacagatgagaaccacc
CDC-10 P1	GTAATACGACTCACTATAGGGCGAATTGGGGCGGCCGCtgaagtatcccagcagttcc
CDC-10 P2	CGATAAGCTTGATATCGAATTCTTACTTGTAcaggcaacctaacctaactgg
CDC-10 P3	GTAATACGACTCACTATAGGGCGAATTGGGGCGGCCGCgtttcgacagcatcacct
CDC-10 P4	GCCTCCGCCTCCGCCTCCGCCGCCTCCGCCgtagccgttcatggtcat
CDC-10 P5	GCTATACGAAGTTATGGATCCGAGCTCGGTACgtggagtggacttcttaacc
CDC10 P6	CAAAAGCTGGAGCTCCACCGCGGTGGCGGCCGCcctcccaagtaggattcatgc
CDC-10 N1	GTAATACGACTCACTATAGGGCGAATTGGGGCGGCCGCgggagggaagttgtttggagaa
CDC-10 N2	GCTATACGAAGTTATGGATCCGAGCTCGGTACccctcaacttcgccgtgc
CDC-10 N3	CGATAAGCTTGATATCGAATTCTTACTTGTAgctgtgtcctgagggtttgg
CDC-10 N4 GFP	GGTGAACAGCTCCTCGCCCTTGCTCACCATgattgcgaacttgatggggg
CDC-10 N4 V5	CCGAGGAGGGGGTTGGGGATGGGCTTGCCCATgattgcgaacttgatggggg
CDC-10 N5	GGCGGAGGCGGCGGAGGCGGAGGCGGAGGCatgtcgaccatgggccct
CDC-10 N6	CAAAAGCTGGAGCTCCACCGCGGTGGCGGCCGCcctccacgttgatcacgcc
CDC-10 CCG1 P9	TTCACAACCCCTCACATCAACCAAATCTAGatgtcgaccatgggccct
CDC-10 P9 IFNTERM	GGAGGCGGCGGAGGCGGCatgtcgaccatgggccct
CDC-10 P2 IF NTERM	TGGCGGCCGCTCTAGActagtagccgttcatggtcatgcg
CDC-11 P1	GTAATACGACTCACTATAGGGCGAATTGGGGCGGCCGCatgacagcagagacttgagc
CDC-11 P2	CGATAAGCTTGATATCGAATTCTTACTTGTAtagaggtgaggtagtgttgg
CDC-11 P3 V2	GTAATACGACTCACTATAGGGCGAATTGGGGCGGCCGCgattaaccattgcattatctcccaggc
CDC-11 P4 V2	GCCTCCGCCTCCGCCTCCGCCGCCTCCGCCgtttccatctggggaaccgttaatttc
CDC-11 P5	GCTATACGAAGTTATGGATCCGAGCTCGGTACacgacactagcacacacatacc
CDC-11 P6	CAAAAGCTGGAGCTCCACCGCGGTGGCGGCCGCcaagtatcatgtggaggagg
CDC-12 P1	GTAATACGACTCACTATAGGGCGAATTGGGGCGGCCGCgaagttcagttacaggctgc
CDC-12 P2	CGATAAGCTTGATATCGAATTCTTACTTGTAgggtagaggattaagtcacg
CDC-12 P3	GTAATACGACTCACTATAGGGCGAATTGGGGCGGCCGCcacaaaggctgagctcgaggag
CDC-12 P4	GCCTCCGCCTCCGCCTCCGCCGCCTCCGCCgcggcgaccgtggctg
CDC-12 P5	GCTATACGAAGTTATGGATCCGAGCTCGGTACaactgggcgacttacatacc
CDC-12 P6	CAAAAGCTGGAGCTCCACCGCGGTGGCGGCCGCcgtacttgtggtcctttagc
ASP-1 P1	GTAATACGACTCACTATAGGGCGAATTGGGGCGGCCGCctatcctcctcttcctttcc
ASP-1 P2	CGATAAGCTTGATATCGAATTCTTACTTGTAagaggtaactaaagccgacc
ASP-1 P3	GTAATACGACTCACTATAGGGCGAATTGGGGCGGCCGCcaagagtgccttggcac
ASP-1 P4	GCCTCCGCCTCCGCCTCCGCCGCCTCCGCCacggatctttgcgcc
ASP-1 P5	GCTATACGAAGTTATGGATCCGAGCTCGGTACccatgttatggagacagagc

ASP-1 P6	CAAAAGCTGGAGCTCCACCGCGGTGGCGGCCGCgatagagacagggacagagg
ASP-1 N1	GTAATACGACTCACTATAGGGCGAATTGGGGCGGCCGCgcggatttggcctggctt
ASP-1 N2	GCTATACGAAGTTATGGATCCGAGCTCGGTACctttggaccgatggaggtgag
ASP-1 N3	CGATAAGCTTGATATCGAATTCTTACTTGTAcgtctatcctcctcttcctttccc
ASP-1 N4 GFP	GGTGAACAGCTCCTCGCCCTTGCTCACCATagctctcggtgagagactgaatgc
ASP-1 N4 V5	CCGAGGAGGGGGTTGGGGATGGGCTTGCCCATagctctcggtgagagactgaatgc
ASP-1 N5	GGCGGAGGCGGCGGAGGCGGAGGCGGAGGCatgaaccagtctcctgctcgc
ASP-1 N6	CAAAAGCTGGAGCTCCACCGCGGTGGCGGCCGCcccagaccgccctctttagc
ASP-1 CCG1 P9	TTCACAACCCCTCACATCAACCAAATCTAGatgaaccagtctcctgctcg
ASP-1 P9 IF NTERM	GGAGGCGGCGGAGGCGGCatgaaccagtctcctgctcg
ASP-1 P2 IF NTERM	TGGCGGCCGCTCTAGActaacggatctttgcgcc
ASP-2 P1	GTAATACGACTCACTATAGGGCGAATTGGGGCGGCCGCgacacatcttggttgacagc
ASP-2 P2	CGATAAGCTTGATATCGAATTCTTACTTGTAggaatgggtagctagtatgg
ASP-2 P3	GTAATACGACTCACTATAGGGCGAATTGGGGCGGCCGCgctgggtggagagggtggt
ASP-2 P4	GCCTCCGCCTCCGCCTCCGCCGCCTCCGCCccgccaaatccgattccac
ASP-2 P5	GCTATACGAAGTTATGGATCCGAGCTCGGTACggttgatcctctgctactgg
ASP-2 P6	CAAAAGCTGGAGCTCCACCGCGGTGGCGGCCGCatcagctgtcgtagtcttgg
ASP-2 N1	GTAATACGACTCACTATAGGGCGAATTGGGGCGGCCGCcgcttccacgtcgatccttt
ASP-2 N2	GCTATACGAAGTTATGGATCCGAGCTCGGTACgattccccgacggtgttactc
ASP-2 N3	CGATAAGCTTGATATCGAATTCTTACTTGTAgacacatcttggttgacagcg
ASP-2 N4 GFP	GGTGAACAGCTCCTCGCCCTTGCTCACCATcgcaaagagagcaatctacatacac
ASP-2 N4 V5	CCGAGGAGGGGGTTGGGGATGGGCTTGCCCATcgcaaagagagcaatctacatacac
ASP-2 N5	GGCGGAGGCGGCGGAGGCGGAGGCGGAGGCatgcgctcaaacaacgatgc
ASP-2 N6	CAAAAGCTGGAGCTCCACCGCGGTGGCGGCCGCgaccttgaaccgcctttagtctg
ASP-2 CCG1 P9	TTCACAACCCCTCACATCAACCAAATCTAGatgcgctcaaacaacgatg
ASP-2 IF NTERM P9	GGAGGCGGCGGAGGCGGCatgcgctcaaacaacgatg
ASP-2 P2 IF NTERM	TGGCGGCCGCTCTAGAtcaccgccaaatccgatt
HPH Y HR FW CC	TACAAGTAAGAATTCGATATCAAGC
HPH YHR RV DD	GTACCGAGCTCGGATCC
NAT Y HR FW CC	TACAAGTAAGAATTCGATATCAAGCTTATCGcaactgatattgaaggagcatttt
NAT Y HR RV DD	GTACCGAGCTCGGATCCATAACTTCGTATAGCcttgtcgacgaattcagatg
NAT Y HR RV YY	CAAAAGCTGGAGCTCCACCGCGGTGGCGGCCGCcttgtcgacgaattcagatg
YRC BB GFP TAGT FW	GGCGGAGGCGGCGGAGGCGGAGGCGGAGGCATGGTG
YRC CC RV	CGATAAGCTTGATATCGAATTCTTACTTGTA
MCHERRY FW BB YRC	GGCGGAGGCGGCGGAGGCGGAGGCGGAGGCatggtgagcaagggcgagg
MCHERRY RV CC YRC	CGATAAGCTTGATATCGAATTCTTACTTGTACAGCTCGTCCATGCC
V5 HAT YRC A FW	GGCGGAGGCGGCGGAGGCGGAGGCGGAGGCGGCAAGCCCATCCCCAACCCCCTCCTCGGAC TCGACAGCACC
V5 HAT YRC B RV V2	CTTGTGGACGTTGTGGATGAGGTGGTCCTTGGCGCCGGCGCCGGCGCCGGTGCTGTCGAGTC CGAGGAGGGGGTTGGG
V5 HAT YRC C FW V2	AAGGACCACCTCATCCACAACGTCCACAAGGAGGAGCACGCCCACGCCCACAACAAGTAAT ACAAGTAAGAATTCGATATCAAGCTTATCG

YRC NT FP START FW	ATGGTGAGCAAGGGCGAGG
YRC NT V5-HAT START FW	ATGGGCAAGCCCATCCCCAAC
YRC NT FP RV BB	GCCTCCGCCTCCGCCTCCGCCGCCTCCGCCcttgtacagctcgtccatgcc
YRC NT V5-HAT RV BB	GCCTCCGCCTCCGCCTCCGCCGCCTCCGCCcttgttgtgggcgtgggc
TAGRFP IF Fw	CCCCGGGTTAATTAAGGGAGCTGGTGCAATGGTGTCTAAGGGCGAAGA
TAGRFP IF Rv	CGATAAGCTTGATATCGAATTCTTACTTGTACAGCTCGTCC
PCCG1 FW YRC XX	GTAATACGACTCACTATAGGGCGAATTGGGGCGGCCGCgctagaaggagcagtccatc
BTUB RV YRC BB	GCCTCCGCCTCCGCCTCCGCCGCCTCCGCCgttaattaactcctcgccctc
CDC-3 G1	TCCCCTTAGCATGTCCTTTT
CDC-3 G2	AAGAGAGTGTTAACGAGGGTC
CDC-3 G3	CTTCTACCTTGAGCGACAACC
CDC-3 G4	GGGGCATAGACAAATTTCCT
CDC-10 G1	CTTCACACATGGACACCTAGA
CDC-10 G2	GGGGTGTCGACAATGTT
CDC-10 G3	GTTTCGACAGCATCACCT
CDC-10 G4	CTTATCCTTCCACCTGGAGT
CDC-11 G1	CTCGGTTACCAAGTGGAATG
CDC-11 G2	TGATGAAGTAGAGCATGGC
CDC-11 G3	CCTTTGTCAACACCCTCTG
CDC-11 G4	CATCTTCGTCTTCCTCACAG
CDC-12 G1	GAACTGGAGGACTCTTTCGAG
CDC-12 G2	AAACCTACCCTGGACTTGAA
CDC-12 G3	GTTCTTCAAGGGTACGACAG
CDC-12 G4	CAGCCATCTCGAGTACATCAT
ASP-1 G1	GTCGTTTGTGATCTCATCGA
ASP-1 G2	GCTAGCATAGCTGTAACGTCC
ASP-1 G3	CAAGAGTGCCTTGGCAC
ASP-1 G4	GCCAACATCTCGAGTACTTTC
ASP-2 G1	CAACACCAGGGATTCGCT
ASP-2 G2	CGACGATGTGTCACTGACA
ASP-2 G3	GACTAAAGGCGGTTCAAGGTC
ASP-2 G4	CCTGAGATCAAATTGCCAACG
MUS-51 Fw	CTTGGAGGAAGGATCAGGAT
MUS-51 Rv	CTCTCAACTAGCTCAGCC
MUS-52 Fw	GACAAGGAAGCCACAGTCTA
MUS-52 Rv	CTCCGAGAGAAGGGCGTC
ACT-1 Fw	GACGACATGGAGAAGATTTGG
ACT-1 Rv	CCTAGCCTATACTGCCATCTCC
HPH 1 Fw	TCACTGGCAAACTGTGATG
HPH 2 Rv	ACACATGGGGATCAGCAATC
NAT 1 Fw	CTTCGTGGTCGTCTCGTA

NAT 2 Rv	CGCCAGGTCGCCGTCG
HIS-3 3' Fw	TTGCCATCTCCACCATCC
HIS-3 5' Rv	GCTGTAGACCAGACCCAGAGC
GFP Fw	CAAGATCCGCCACAACATCG
GFP Rv	ATGTGGTCGGGGTAGCGG
